# Single-cell lineage tracing identifies hemogenic endothelial cells in the adult mouse bone marrow

**DOI:** 10.1101/2025.10.09.681472

**Published:** 2025-10-10

**Authors:** Jing-Xin Feng, Mei-Ting Yang, Lili Li, Caiyi C. Li, Ferenc Livák, Jack Chen, Yongmei Zhao, Dunrui Wang, Avinash Bhandoola, Naomi Taylor, Giovanna Tosato

**Affiliations:** 1 Laboratory of Cellular Oncology, Center for Cancer Research, National Cancer Institute (NCI), National Institutes of Health, Bethesda, MD, 20892, USA; 2 School of Life Sciences, Northeast Normal University, Changchun, China; 3 Experimental Immunology Branch, Center for Cancer Research (CCR), National Cancer Institute (NCI), National Institutes of Health, Bethesda, MD, 20892, USA; 4 Laboratory of Genome Integrity, Center for Cancer Research, National Cancer Institute (NCI), National Institutes of Health, Bethesda, MD, 20892, USA; 5 Sequencing Facility Bioinformatics Group, Bioinformatics and Computational Science Directorate, Frederick National Laboratory for Cancer Research, Frederick, MD, 21702, USA; 6 Pediatric Oncology Branch, Center for Cancer Research (CCR), National Cancer Institute (NCI), National Institutes of Health, Bethesda, MD, 20892, USA.; 7 Senior author; 8 Lead contact

**Keywords:** Hematopoietic stem and progenitor cells, endothelial cells, hematopoiesis, endothelial to hemogenic transition, mesenchymal cells, blood, *Runx1*, *Col1a2*, single cell lineage tracing, single cell RNA sequencing

## Abstract

During mouse development, hematopoietic stem and progenitor cells (HSPC) originate from hemogenic endothelial cells (ECs) through a process of endothelial-to-hematopoietic transition. These HSPC are thought to fully sustain adult hematopoiesis. However, it remains unknown whether adult ECs retain hemogenic potential. Here we used in vivo genetic lineage tracking at population and single-cell (sc) levels, scRNA sequencing, and bone marrow transplantation to detect hemogenic ECs in adult mice. We identify and characterize a small population of bone marrow-resident, adult *Cdh5*/VE-Cadherin^+^ ECs that produce hematopoietic cell-progeny in vitro and in mice. These adult hemogenic ECs and their hematopoietic cell progeny can give rise to hematopoietic cells following adoptive transfer into adult mice. Furthermore, blood cells generated from adult and developmental ECs comparably home to peripheral tissues, where they similarly contribute to inflammatory responses. Thus, our results identify previously unrecognized bone marrow-derived adult hemogenic ECs that generate HSPC and functional mature blood cells.

## INTRODUCTION

During development, hemogenic ECs generate hematopoietic cells through a process of endothelial-to-hematopoietic transition (EHT) at geographically defined anatomical sites^1–9^. At these locations, the hemogenic ECs represent a small fraction of all ECs^10–12^, and their competency to produce hematopoietic stem and progenitor cells (HSPC) is temporally restricted to short developmental windows, and the hemogenic potential differs^13–18^. In the dorsal aorta, the hemogenic endothelium produces hematopoietic stem cells (HSC) and other multi-potent progenitors between E10.5 and E11.5^17-19^ that persist in the adult and are generally believed to sustain adult hematopoiesis throughout life.

Efforts to reliably generate *ex vivo* HSC from Cdh5-expressing ECs identified difficulties in reproducing the microenvironmental clues coming from the inductive niche cells^20–22^ and have generally relied on transcription factor-induced reprogramming of the ECs to drive hematopoietic specification^23,24^. The transcription factor RUNX1, which marks the hemogenic endothelium, can confer a hemogenic potential to embryonic ECs lacking such potential^25–28^. When transcription factors RUNX1, FOSB, GFI1, and SPI1 were co-expressed in adult murine ECs co-cultured with “vascular niche ECs”, EHT was induced in vitro producing HSC with long-term self-renewal capacity^29^. A similar approach was used with human ECs enabling hematopoietic specification^30^.

An unresolved question is whether hemogenic ECs, thought to be mostly restricted to the early stages of mouse development^12,23^, may persist in the adult mouse^2,12,31,32^. Exploiting advances in cell lineage tracking and single cell analyses^33,34^, we report the identification of hemogenic ECs in the adult mouse bone marrow (BM) that produce functional hematopoietic progenitors and mature blood cells.

## RESULTS

### Evidence that adult bone marrow ECs generate hematopoietic cells

**Figure 1.**
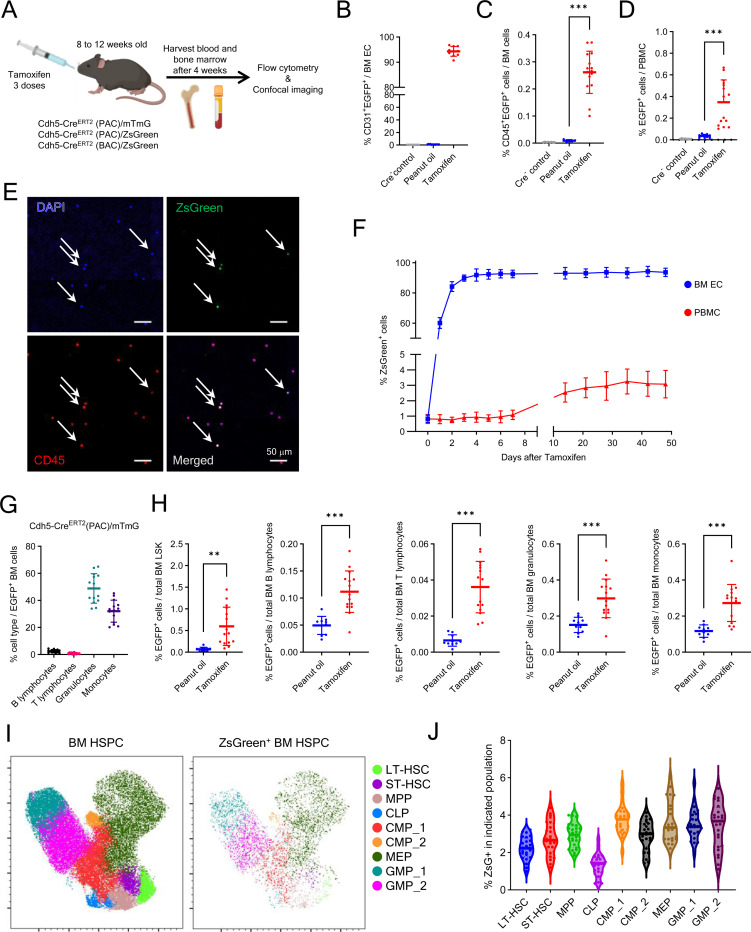
Lineage tracking discloses a contribution of endothelial cells to hematopoiesis in adult BM (A) Experimental design: tamoxifen was administered to 8- to12-week-old Cdh5-Cre mice to induce fluorescent labeling of VE-Cadherin^+^ cells and their cell progeny. Four weeks later, BM and blood were analyzed. (B) CD31^+^EGFP^+^ BM ECs in Cre^−^ mice (n=10) and Cre^+^ mice treated with oil (n=13) or tamoxifen (n=10); flow cytometry results. (C and D) CD45^+^EGFP^+^ cells in BM and blood from Cre^−^ mice (n=8) and Cre^+^ mice treated with oil (n=6–10) or tamoxifen (n=15–18). Representative flow cytometry gating in Figure S1G. (E) Representative blood smear from a tamoxifen-treated Cdh5-Cre^ERT2^(PAC)/ZsGreen mouse showing ZsGreen^+^CD45^+^DAPI^+^ cells (arrows). (F) Kinetics of ZsGreen^+^ cell detection in BM ECs (CD45^−^VE-Cadherin^+^) and blood white blood cells (WBC) post-tamoxifen; mouse n=8–10/group). (G) EGFP^+^ B and T-lymphocytes, granulocytes, and monocytes in BM of tamoxifen-treated mice (n=14) as percent of total EGFP^+^ cells; 3 experiments. (H) EGFP^+^ BM LSK, lymphocytes, granulocytes, and monocytes as percent of total EGFP^+/−^ cell type; Cdh5-Cre^ERT2^(PAC)/mTmG mice (oil n=10; tamoxifen n=15), 3 experiments. (I) UMAP plots of Lin^−^ BM HSPC from tamoxifen-treated Cdh5-Cre^ERT2^(PAC)/ZsGreen mice (n=26; 1 femur/mouse) showing FlowSOM clustering of all (ZsGreen^+/−^) and ZsGreen^+^ populations. (J) Violin plots showing ZsGreen^+^ cell distribution across HSPC subsets from (I). Dots represent individual mice; data shown as mean±SD except shown as median in (G). *p < 0.05, **p < 0.01, ***p < 0.001 by Student’s *t* test.

We assessed the hemogenic potential of ECs in adult mice using Cre-reporter-based lineage tracing. Since *Cdh5*, encoding vascular endothelial cadherin (VE-Cadherin), is selectively expressed by ECs, Cdh5-Cre^ERT2^ recombinase activity allows tracking the hematopoietic cell output from adult hemogenic ECs^35,36^. Therefore, we generated three Cdh5-based lineage-tracing models using inducible Cdh5-Cre^ERT2^(PAC)^37,38^ and Cdh5-Cre^ERT2^(BAC)^39^ mouse lines, in combination with the Cre-reporter lines ZsGreen and mTmG (Figure S1A). We then treated eight- to twelve-week-old mice with three doses of tamoxifen (10 mg∙kg^-1^, gavage) on consecutive days, and four weeks later we evaluated peripheral blood and BM (Figure 1A). Expectedly^36^, most BM Endomucin^+^ ECs were ZsGreen^+^ in tamoxifen-treated Cdh5-Cre^ERT2^(PAC)/ZsGreen and Cdh5-Cre^ERT2^(BAC)/ZsGreen mice, and virtually no ZsGreen^+^ ECs were present in Cre negative or peanut oil-treated controls (Figures S1B and S1C). By flow cytometry, >90% CD31^+^VE-Cadherin^+^ BM ECs were tracked by tamoxifen-induced fluorescence in the three mouse lines (Figure 1B; Figure S1D). A low-level tamoxifen-independent reporter fluorescence was also detected in BM ECs, which was low in the mTmG reporter line (Figure 1B), and higher in the ZsGreen reporter lines, previously attributed to “basal” Cre^ERT2^ activity^40^ (Figure S1D).

To evaluate the hemogenic potential of adult BM ECs, we analyzed the expression of the hematopoietic marker CD45 in Cdh5-tracked cells. Notably, tracked CD45^+^ hematopoietic cells, presumed progeny of VE-Cadherin^+^ ECs, were detected by flow cytometry in BM and blood of mice from all three Cdh5-Cre^ERT2^ mouse lines (Figure 1C and D; Figures S1E and F; representative flow cytometry gating in Figure S1G and H). Moreover, confocal microscopy identified isolated ZsGreen^+^CD45^+^ cells in BM (Figure S1I) and blood of tamoxifen-induced mice (Figure 1E). Importantly, while a small fraction of fluorescent CD45^+^ cells were detected in EGFP and ZsGreen (Figures 1C and D; and Figure S1D-F) reporter mice without tamoxifen, as reported^40–43^, the significant increase of tamoxifen-induced fluorescent CD45^+^ cells (Figure 1C and 1D, Figure S1D-F) indicates the occurrence of Cdh5-Cre recombination in the adult mouse, presumably tracking EHT. This tamoxifen-induced increase in CD45^+^ZsGreen^+^ peripheral blood mononuclear cells (PBMC) was also observed in individual mice (Figure S1J).

We tested the kinetics of tamoxifen-induced ZsGreen expression in BM ECs and PBMC of Cdh5-Cre^ERT2^(PAC)/ZsGreen mice (Figure 1F). By flow cytometry, virtually all BM ECs were ZsGreen^+^ by day 4 after tamoxifen administration and this level persisted over 50 days. Instead, the percentage of ZsGreen^+^ PBMCs increased more gradually, plateauing by day 14 and this level persisted over 50 days of observation. This gradual increase is likely attributable to tamoxifen-induced ZsGreen expression in hemogenic ECs.

Further analysis showed that most BM EGFP^+^CD45^+^ cells in Cdh5-Cre^ERT2^(PAC)/mTmG mice were CD11b^+^Ly6G^+^ granulocytes (48.85%) and CD11b^+^Ly6G^−^ monocytes (32.06%), while CD19^+^ B (2.46%) and CD3^+^ T (0.84%) lymphocytes were detected at lower frequencies (Figure 1G). All EGFP^+^ BM cell populations were significantly induced by tamoxifen administration, including rare LSK (Lin^-^Sca1^+^cKit^+^) progenitors (Figure 1H). Also, the peripheral blood of tamoxifen-induced Cdh5-Cre^ERT2^(PAC)/mTmG mice contained EGFP^+^ granulocytes and monocytes, and fewer B and T lymphocytes (Figure S1K and L). Similarly, in ZsGreen-reporter mice, tracked LSK progenitors, B lymphocytes, granulocytes, and monocytes were all present in the BM and blood (Figure S2A-D).

We further characterized the tracked BM hematopoietic LSK stem/progenitors by flow cytometry (Figure S2E). The results, displayed by Uniform Manifold Approximation and Projection (UMAP) dimensional reduction, showed that the ZsGreen-tracked progenitor population includes cell subsets defined phenotypically as hematopoietic stem and progenitor cells (HSPC) (Figure 1I). Each ZsGreen^+^ progenitor population represented a similarly small proportion of the corresponding non-tracked progenitor cell population (Figure 1J). These results support the existence of EHT in the adult mouse contributing to generation of hematopoietic progenitors and mature cells.

### Adult BM ECs cultured ex vivo generate transplantable HSPCs

**Figure 2.**
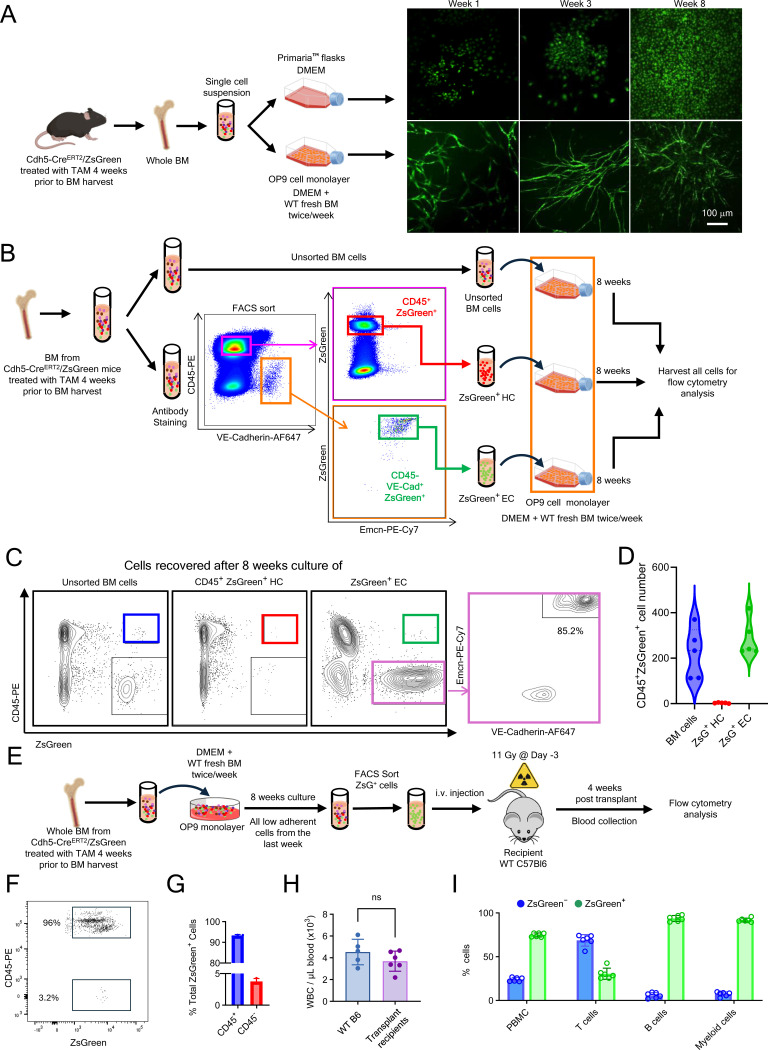
BM ECs generate engraftable hematopoietic cells ex vivo (A) BM cells from tamoxifen-treated mice were cultured on high-attachment Primaria flasks or OP9 cell monolayers. Representative images show ZsGreen^+^ cells at weeks 1, 3, and 8. (B) Workflow for culturing unsorted and sorted BM cell populations. All cells were cultured (8 weeks) on OP9 cell monolayers supplemented with WT BM cells. Culture medium and floating cells were removed twice/week for 7 weeks. At the start of week 8, one final WT BM and medium supplementation was implemented prior to harvest at the end of week 8. Representative image (bottom left) shows sorted ZsGreen^+^ ECs on OP9 monolayer after 4 weeks of culture. (C and D) Representative flow cytometry plots (C) and quantification (D) of CD45^+^ZsGreen^+^ cells from each of the 8-week cultures (n=5). (E) Floating/loosely adherent ZsGreen^+^ cells from unsorted BM 8-week cell cultures were sorted and transplanted (5×10^4^, 2.5×10^4^,1.25×10^4^ or 6.25×10^3^ cells) into lethally irradiated (11 Gy) WT mice (n=2/group). (F - G) Representative image (F) and quantification (G) of low-adherent cells harvested after 8 weeks of culture, showing that >95% of ZsGreen^+^ low-adherent cells are CD45^+^. (H and I) WBC counts (F) and percent ZsGreen^+^ and ZsGreen^−^ cells (G) in blood of transplant recipients 10 weeks post-transplant (n=6); WT controls (no irradiation or transplant; n=5). Dots represent individual mice. Data are shown as mean ± SD. ns, not significant by Student’s *t* test.

To investigate whether adult BM ECs can generate hematopoietic cells *ex vivo*, we cultured BM cells isolated from tamoxifen-treated Cdh5-Cre/ZsGreen mice. Initially, BM single-cell (sc) suspensions were cultured under two conditions (Figure 2A): (1) on “Primaria” pro-adhesive flasks, and (2) on OP9 stromal cell^44^ monolayers grown on gelatin-coated conventional tissue culture flasks. To attempt recreating BM niches, fresh wild-type (WT) BM cells were added to the ZsGreen-tracked BM cell cultures twice/week throughout the culture period (see Methods for details). By week 8, BM cells cultured on “Primaria” dishes formed a confluent monolayer of ZsGreen-tracked cells with a typical EC morphology, whereas BM cells cultured on OP9 monolayers exhibited a fibroblast-like morphology with the ZsGreen^+^ cells clustering in foci, without forming a monolayer (Figure 2A). We visualized some ZsGreen-tracked (ZsGreen^+^) cells in cultures of BM cells grown onto OP9 monolayers supplemented with fresh BM cells, but not in cultures of BM cells grown onto “Primaria” surfaces supplemented with fresh BM cells (Figure S2F). This hinted that the OP9 and BM cell culture system may allow hematopoietic cells emergence from Cdh5-Cre/ZsGreen-tracked EC ex vivo.

To test this possibility, we generated a pool of BM cells (from 20 adult mice; age 10–18 weeks) and used the pool to derive 3 populations: sorted BM CD45^−^VE-Cadherin^+^ZsGreen^+^ ECs; sorted CD45^+^ZsGreen^+^ hematopoietic cells; and unsorted BM cell populations (Figure 2B). We then cultured these 3 cell populations under the same conditions (OP9 monolater and supplementation with fresh BM cells twice/week). After 8-week culture, flow cytometry analysis detected CD45^+^ZsGreen^+^ cells from the unsorted BM and the sorted CD45^−^VE-Cadherin^+^ZsGreen^+^ cell cultures, but the CD45^+^ZsGreen^+^ were virtually absent from the CD45^+^ZsGreen^+^ cell cultures (Figure 2C and 2D). These results show that CD45^−^VE-Cadherin^+^ZsGreen^+^ ECs can generate CD45^+^ZsGreen^+^ *ex vivo*. Notably, most (>80%) CD45^−^ZsGreen^+^ cells retained expression of VE-Cadherin and Endomucin, thereby confirming their endothelial identity (Figure 2C, most right panel). Importantly, the virtual absence of CD45^+^ZsGreen^+^ cells in 8-week cultures of sorted CD45^+^ZsGreen^+^ cells shows that pre-existing CD45^+^ZsGreen^+^ hematopoietic cells, derived from tamoxifen-dependent and independent processes, are effectively removed during the extended culture. This further suggests that the CD45^+^ZsGreen^+^ hematopoietic cells detected in the CD45^−^VE-Cadherin^+^ZsGreen^+^ EC cultures likely originated from ECs during the final week of culture.

Next, we tested whether the *ex vivo*-derived CD45^+^ZsGreen^+^ cells are functional in lethally irradiated recipients. To this end, we obtained unfractionated BM cells from 50 tamoxifen-treated Cdh5-Cre/ZsGreen mice, cultured the cells onto OP9 monolayers with fresh BM supplementation, sorted the ZsGreen^+^ cells (96% of which were CD45^+^ by flow cytometry) at the end of 8-week culture, and transplanted these cells into 8 lethally irradiated WT recipients; 5×10^3^ cells; 2.5×10^3^ cells; 1.25×10^3^ cells; 6.25×10^2^ cells, n=2/group (Figure 2E-G). Two mice (recipients of 6.25×10^2^ and 1.25×10^3^ cells) died on days 4 and 6, but the remaining six mice survived and remain well at the time of manuscript submission (12 weeks post-transplant). Ten weeks post-transplant, peripheral blood WBC counts were within normal range in all surviving transplant recipients, indicative of hematopoietic reconstitution (Figure 2H). Flow cytometry revealed that 74.85% of PBMC were ZsGreen^+^ (Figure 2I). Most myeloid cells (granulocytes and monocytes, 91.97%) and B cells (93.78%) were ZsGreen^+^, whereas only 30.63% of T cells were ZsGreen^+^, likely attributable to long-lived ZsGreen^−^ T cells.

These results confirm that BM ECs propagated *ex vivo* onto OP9 monolayers with BM cell supplementation produce hematopoietic cells and show that this output includes HSPC capable of engrafting and generating hematopoietic cell progeny of myeloid and lymphoid lineages in lethally irradiated recipients.

### Adult BM ECs can give rise to hematopoietic cells after transfer to conditioned recipients

**Figure 3.**
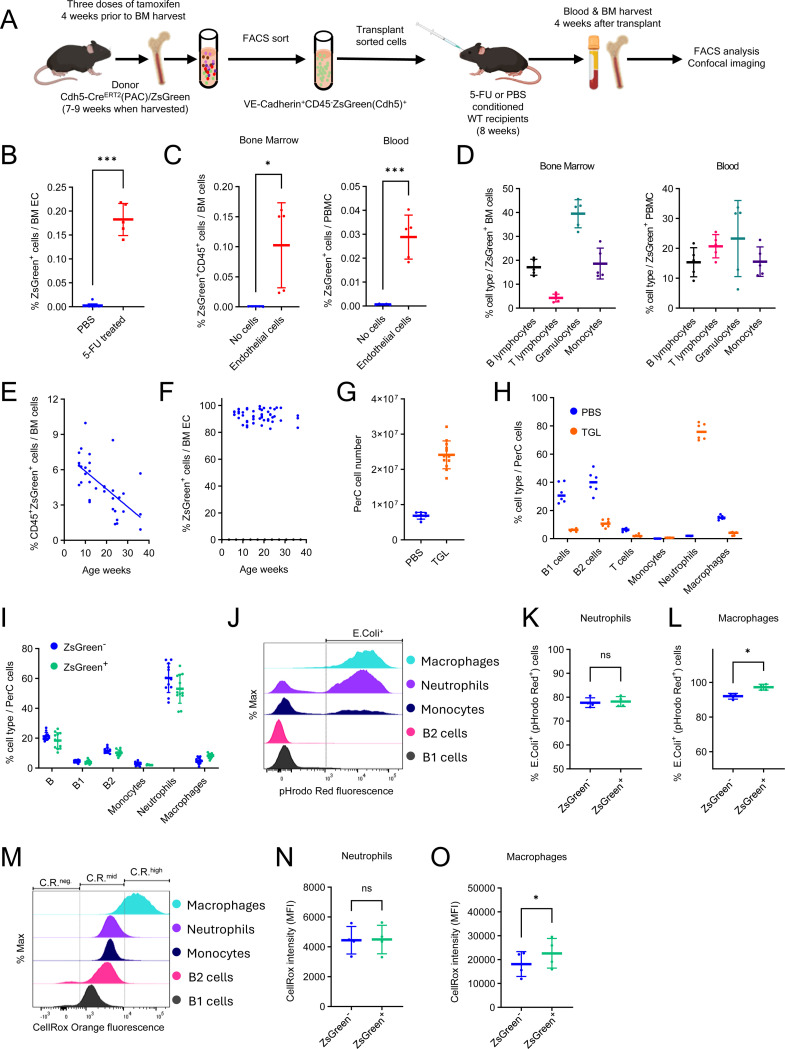
Adult BM endothelial cells give rise to hematopoietic cells following transfer into conditioned recipients (A) Transplant experiment: donor ECs from BM of tamoxifen-treated mice were FACS-sorted and transplanted into WT C57Bl/6 recipients conditioned with 5-FU or PBS. (B) ZsGreen^+^ ECs detected in BM of 5-FU-conditioned (n=5) or PBS-conditioned (n=15) recipients of ECs 4 weeks post-transplant. (C and D) ZsGreen^+^CD45^+^ cells (C) and cell type distribution (D) in the BM and blood of 5-FU-conditioned transplant recipients of BM ECs or no cell controls (n=5/group). (E and F) Age-dependent decline of ZsGreen^+^CD45^+^ cells (E) but not ZsGreen^+^VE-Cadhenin^+^ cells (F) in the BM of Cdh5-Cre^ERT2^(BAC)/ZsGreen mice (n=35) treated with tamoxifen 4-weeks prior to harvest. (G and H) Cell number (G; mouse n=8–12) and cell type distribution (H; mouse n=6) in the peritoneal cavity (PerC) of PBS- or thioglycolate (TGL)-pretreated (4 hours) mice. (I) ZsGreen^+^ and ZsGreen^−^ PerC cell types in TGL-pretreated mice (n=12). (J) Representative histograms depicting pHrodo Red fluorescence detection of E-Coli phagocytosis. (K and L) E. coli^+^ phagocytosis by ZsGreen^+^ and ZsGreen^−^ PerC neutrophils (K) and macrophages (L) in TGL-pretreated mice (n=4). (M) Representative histograms depicting CellRox Orange fluorescence for cell-associated ROS detection. (N and O) CellRox mean fluorescence intensity (MFI) in ZsGreen^+^ and ZsGreen^−^ PerC neutrophils (N) and macrophages (O) in TGL-pretreated mice (n=4). Dots represent individual mice. Data are shown as mean ± SD. *p < 0.05, ***p < 0.001, ns, not significant by Student’s *t* test.

Next, we examined whether adult BM ECs are hemogenic in transplant recipients. To this end, we FACS-sorted CD45^-^VE-Cadherin^+^ZsGreen^+^ ECs from the BM of tamoxifen-pretreated (4 weeks prior to BM harvest) Cdh5-Cre^ERT2^(PAC)/ZsGreen mice (Figure 3A; Figure S2G and S2H) and transplanted these cells into adult WT C57Bl/6 mice untreated (PBS) or conditioned by fluorouracil (5-FU treated) prior to transplant^45^. The choice of 5-FU conditioning rather than lethal irradiation of adult mice was driven by previous experiments showing the difficulties at reconstituting lethally myelo-ablated adult recipients with hemogenic yolk sac cells, which reconstituted conditioned newborns^46^. Four weeks after transplant, we observed a significant increase in the proportion of ZsGreen^+^ ECs in the BM and ZsGreen^+^CD45^+^hematopoietic cells in the BM and peripheral blood of the transplant recipients conditioned by 5-FU but not controls (PBS) (Figure 3B and 3C; Figure S2I). These CD45^+^ZsGreen-tracked cells in BM and blood included granulocytes, monocytes, and lymphocytes (Figure 3D). Thus, BM-derived CD45^−^VE-Cadherin^+^ZsGreen^+^ cells, when transferred into 5-FU-conditioned recipients, gave rise to detectable CD45^+^ZsGreen^+^ hematopoietic cells.

We further examined the effect of mouse age on the endothelial hemogenic potential by treating the mice with tamoxifen between week 6 and 32 of age. Four weeks later (week 10 to 36 of age), we measured the percentage of ZsGreen-tracked CD45^+^ hematopoietic cells in the BM. We observed that tamoxifen inductions beyond week 10 of age resulted in a progressive decrease of the CD45^+^ hematopoietic cell output, and detected an inverse correlation (Pearson’s r −0.63, *P*<0.0001) between age and EC hemogenic potential (Figure 3E). This progressive decline of CD45^+^ cell output was not coupled with a loss of BM EC fluorescence, since virtually all BM ECs were ZsGreen^+^ throughout the duration of the experiment (Figure 3F), consistent with the stability of Cre-mediated labeling of Cdh5-expressing cells ^36^. These observations suggest an age-related loss of hemogenic capability of BM ECs.

Additionally, we examined whether tracked hematopoietic cells from adult BM EC are functional. Since lymphocyte trafficking from the peripheral blood to the peritoneal cavity is critical for their function at this site^47,48^, we first evaluated the spontaneous migration of tracked CD45^+^ hematopoietic cells to the peritoneal cavity. Compared to no-tamoxifen controls, tamoxifen-treated Cdh5-Cre^ERT2^(PAC)/ZsGreen mice displayed a significant increase of CD45^+^ZsGreen^+^ monocytes, macrophages, and B1, B2, and T lymphocytes in the peritoneal cavity (Figure S2J and S2K).

After inducing peritonitis with thioglycolate (TGL, 4 hours) in tamoxifen-treated Cdh5-Cre^ERT2^(PAC)/ZsGreen mice, the overall number of peritoneal leukocytes increased substantially compared to untreated (PBS) controls (Figure 3G), mostly attributable to neutrophils (Figure 3H). In addition, the peritoneal ZsGreen^+^ and ZsGreen^−^ cell populations exhibited a similar cell type distribution in TGL-treated mice (Figure 3I). We further examined *Escherichia coli* (K-12 strain) phagocytosis and reactive oxygen species (ROS) production in peritoneal cell exudates in response to TGL (Figure 3J–3O). Both ZsGreen^+^ and ZsGreen^−^ peritoneal neutrophils and macrophages comparably phagocytosed *Escherichia coli* and generated ROS (Figure 3J, 3K, 3M, and 3N), except that the phagocytic and ROS production of ZsGreen^+^ macrophages was somewhat higher than that of ZsGreen^−^ macrophages (Figure 3L and 3O).

Thus, Cdh5-tracked mature neutrophils and macrophages are functional at trafficking and homing, and presumably capable of contributing to the host response to tissue inflammation.

### Adult EHT is independent of preexisting hematopoietic cell progenitors

Faithful Cre-reporter lineage tracing requires that Cre recombinase activity be restricted to the intended cell type^40^. In our system, Cdh5-Cre^ERT2^ is expected to drive recombination specifically in ECs, as *Cdh5* is an established EC-specific marker. However, flow cytometry has occasionally revealed rare VE-Cadherin^+^CD45^+^ in mouse BM, exemplified in Figure S2G, potentially reflecting double-positive cells. To address the possibility that cells co-expressing VE-Cadherin/*Cdh5* and CD45/*Ptprc* exist in the mouse BM, we analyzed publicly available sc-RNAseq data from adult mouse BM^49^. This analysis showed that only a small subset of plasmacytoid dendritic cells (pDCs) co-express VE-Cadherin and CD45, but not HSPC or other mature blood cells (Figure S3A-S3D). pDCs are terminally differentiated cells and unlikely progenitors of tracked CD45^+^ multilineage progeny in our Cdh5-cre mice. Nonetheless, it is plausible that other, currently unidentified, hematopoietic cells may also co-express *Cdh5*/VE-Cadherin and *Ptprc*/CD45. We additionally considered the possibility that CD45^+^ progenitors with functional Cre^ERT2^ activity may contaminate the sorted populations of Cdh5^+^CD45^−^ BM cells and could become fluorescent upon tamoxifen administration.

**Figure 4.**
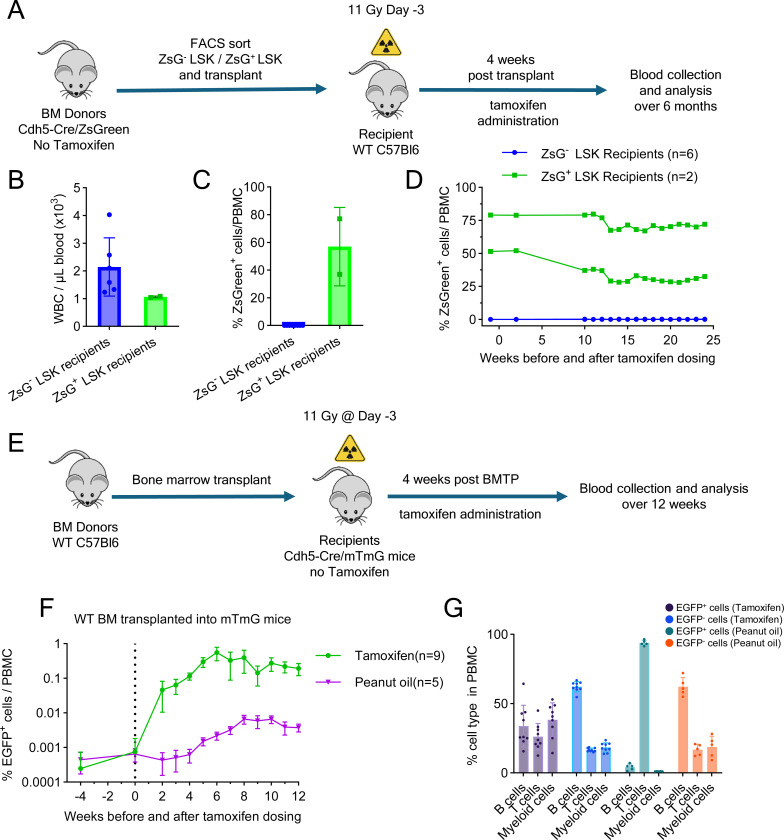
Independence of adult EHT from preexisting HSPC (A) Transplantation experiment: donor LSK sorting, recipient irradiation, transplantation, tamoxifen treatment, and analysis. (B-D) Blood WBC counts (B), percent ZsGreen^+^ PBMC (C), and time course of ZsGreen^+^ PBMC detection (D) in transplant recipients of ZsGreen^−^ LSK (5×10^4^ or 2.5×10^4^ cells/mouse; n=3/group) and ZsGreen-enriched LSKs (2.8×10^3^ cells/mouse; n=2). Results in B and C are from week 24 post-tamoxifen. (E) Experiment: WT BM transplantation (BMTP) into lethally irradiated Cdh5-Cre/mTmG mice (n=9). Four weeks later, tamoxifen was administered; blood was monitored for 16 weeks. (F and G) EGFP^+^ PBMC detection before and after tamoxifen or peanut oil administration (F) and cell type distribution of EGFP^+^ and EGFP^−^ PBMCs at week 12 post-tamoxifen or peanut oil (G) in Cdh5-Cre/mTmG recipients (n=9) of WT BM (5×10^6^ cells). Dots represent individual mice. Data are shown as mean ± SD.

To address both possibilities, we performed transplant experiments with two populations of Lin^−^Sca1^+^cKit^+^ (LSK) progenitors from tamoxifen untreated Cdh5-Cre^ERT2^(PAC)/ZsGreen mice: (1) a ZsGreen^−^ LSK population (>99% purity; Figure S3E-S3G) to assess whether ZsGreen^−^ hematopoietic progenitors with a functional Cdh5-Cre^ERT2^ activity could become fluorescent after tamoxifen, and (2) an LSK population enriched for ZsGreen^+^ cells (45.9% purity, with the remaining 54.1% comprising ZsGreen^−^ LSK cells (Figure S3F)), to assess the contribution of pre-tracked, tamoxifen-independent, hematopoietic progenitors. We then transplanted these two LSK populations into lethally irradiated (11 Gy) WT C57Bl/6 recipients (Figure 4A). Four weeks post-transplant, we administered tamoxifen and monitored the peripheral blood for the presence of ZsGreen^+^ hematopoietic cells over six months.

Expectedly, all LSK recipients (ZsGreen^−^ LSKs or LSK enriched with ZsGreen^+^ cells) showed successful hematopoietic reconstitution as evidenced by normal blood WBC counts by 10 weeks post-transplant (Figure 4B). Importantly, the mice transplanted with ZsGreen^−^ LSKs did not produce ZsGreen^+^ PBMCs post-tamoxifen administration, indicating that the transplanted LSKs and their progeny did not express tamoxifen inducible Cdh5-Cre^ERT2^ recombinase activity (Figure 4C and 4D). Instead, the mice transplanted with ZsGreen^+^-enriched LSKs displayed a similar proportion of ZsGreen^+^ PBMCs prior to and after tamoxifen administration (Figure 4D), indicating that the ZsGreen^+^ LSK has not expanded after tamoxifen administration. Collectively, these results demonstrate that LSK progenitors in adult BM lack of tamoxifen-inducible Cdh5 expression and do not contribute to tamoxifen-induced adult EHT in our Cdh5-reporter mice. Rather, these results strongly support the conclusion that Cdh5^+^CD45^−^ ECs are a source of hematopoietic cells in the adult mouse.

In additional experiments, we took advantage of the relative insensitivity of BM ECs subsets to irradiation relative to BM hematopoietic cells^50^ to examine the possibility that EC surviving after lethal irradiation may be hemogenic. As a lethal dose of irradiation effectively eliminates HSPCs and requires hematopoietic reconstitution for survival, we transplanted WT (untracked) BM cells into lethally irradiated Cdh5-Cre/mTmG mice and treated the mice with tamoxifen 4 weeks after transplantation (Figure 4E). Prior to tamoxifen administration, >99% of PBMCs were not fluorescent, indicating that these cells derived from the transplanted WT BM rather than host-derived (Figure 4F). After tamoxifen treatment, a progressive increase in EGFP^+^ PBMCs was observed, reaching ~0.55% by six weeks, which included myeloid cells and B and T lymphocytes (Figure 4F and 4G). Although we cannot exclude the possibility that rare EGFP^+^ hematopoietic progenitor (tracked tamoxifen-dependently or independently) may have survived the irradiation, the presence of EGFP^+^ hematopoietic cells in the circulation of lethally irradiated Cdh5-Cre/mTmG mice suggests their derivation from radioresistant Cdh5^+^ ECs rather than from radiosensitive hematopoietic progenitors. These results further support the view that adult ECs possess hemogenic potential and can produce hematopoietic cells in vivo.

### Single cell tracking confirms the presence hemogenic ECs in adult BM

**Figure 5.**
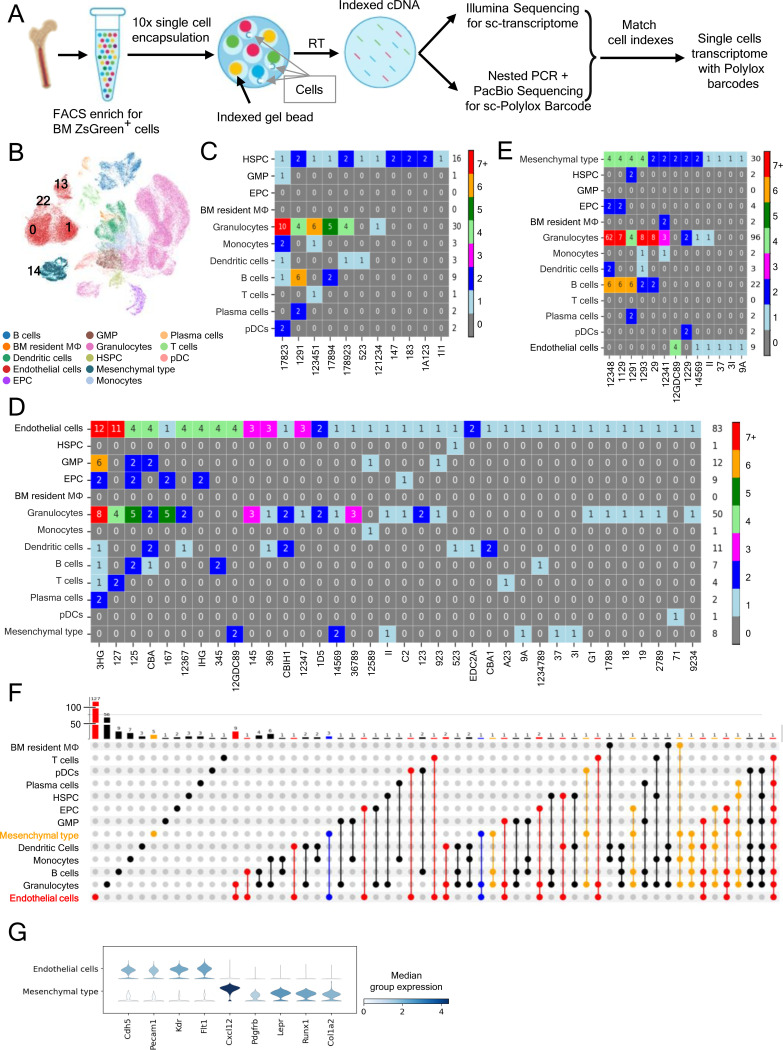
Polylox sc lineage tracing links adult BM ECs to hematopoietic progenitors and mature blood cell progeny (A) Schematic of Polylox barcode and transcriptome profiling. FACS-enriched ECs (ZsGreen^+^VE-Cadherin^+^Endomucin^+^) and EC-depleted (ZsGreen^−^VE-Cadherin^−^Endomucin^−^) BM cells from tamoxifen-treated Cdh5-CreERT2/ZsGreen/PolyloxExpress mice (n=3, 10 week-old at the time of tamoxifen treatment) were mixed (1:1), and encapsulated (147,446 cells loaded; 93,553 processed). Indexed cDNA was used for scRNA-seq and barcode detection by PacBio sequencing after nested PCR enrichment; barcode-transcriptome integration was accomplished via shared cell indices. (B) UMAP clustering and cell type annotation. Clusters 0, 1, 13, and 22 comprise ECs; cluster 14 comprises Mesenchymal-type cells. (C – E) Heatmaps showing “true” Polylox barcodes (pGen < 1×10^−4^) linking HSPCs to hematopoietic cells (C), ECs to hematopoietic and other cells (D), and Mesenchymal-type cells to other cells (E). The numbers within the colored boxes identify cell number; the labels at the bottom of each column denote the barcode shared by all cells in that column; the number on the right side the heatmaps reflects the total number of cells in each row. (F) UpSet plot showing cells (identified by colored dots) sharing the same “true” barcode (identified by lines connecting the colored dots); bar graph at the top of the plot reflects (height and number on each bar) the number of “true” barcodes. Colors of dots: EC (red), Mesenchymal-type (orange), ECs connecting with Mesenchymal-type cells (blue), cells other than ECs and Mesenchymal-type cells (black). (G) Violin plots showing selected gene expression profile in Mesenchymal-type cells (cluster 14) and ECs (clusters 0, 1, 13, 22 combined).

To directly trace hematopoietic cell progeny arising from individual adult ECs, we exploited the *PolyloxExpress* sc genetic barcoding system. We generated Cdh5-Cre^ERT2^/ZsGreen/*PolyloxExpress* mice, in which both the ZsGreen and Polylox transgenes are inserted in the Rosa26 locus, such that individual ZsGreen^+^ cells contain a single Polylox barcode^33,51,52^. To evaluate EC-derived hematopoietic cell output, we harvested BM from tamoxifen-treated Cdh5-Cre^ERT2^/ZsGreen/*PolyloxExpress* mice (n=3), sorted the ZsGreen^+^VE-Cadherin^+^Endomucin^+^ (purity >99 %) and the ZsGreen^+^VE-Cadherin^-^Endomucin^-^CD45^+^ hematopoietic cells (purity >99%), mixed these populations (1:1 ratio; total 147,446 cells) and processed for 10x Illumina Sequencing and PacBio sequencing (detailed in Methods). We recovered sc transcriptome from 93,553 cells; of these, 4,072 cells had a barcode (Figure 5A, Figure S5A, detailed in Methods).

Unsupervised clustering of sc transcriptome data revealed 34 clusters, 31 of which remained after doublet removal (Figure 5B; Figure S5A-S5D). Cell clusters 0, 1, 22, and 13 were annotated as “Endothelial cells” based on expression of the classical EC markers *Cdh5*, *Pecam1*, *Kdr* and *Flt1*, and absence of *Ptprc*/CD45, *Runx1*, and the mesenchymal cell markers *Cxcl12*, *Lepr*, *Pdgfrb*, and *Col1a2* expression (Figure 5B; Figure S4E). Cell cluster 14 was annotated as “Mesenchymal type” based on co-expression of *Cxcl12*, *Lepr*, *Pdgfrb*, *Col1a2*, but expressed *Runx1* and the EC markers *Cdh5* and *Pecam1* (Figure 5B; Figure S5E). The remaining cell clusters included the hematopoietic progenitors and mature blood cells of various lineages (Figure 5B; Figure S4E).

Barcode analysis revealed robust *Polylox* barcode diversity among cell populations, including 296 “true” barcodes, defined as barcodes with low generation probability (*P*_gen_ <1×10^−4^)^51^ consistent with rare, unique recombination events (Figure S5A and S5B). Expectedly, “true” barcodes linked HSPC to downstream progenitors and mature hematopoietic cells, validating the system (Figure 5C).

Notably, 83 ECs (from “endothelial” cell clusters 0, 1, 22, and 13) were marked by “true” barcodes, 31 of which linked EC to hematopoietic cells based on their shared barcode (Figure 5D). These hematopoietic cells linked to ECs by shared barcodes included HSPC, EPC, GMP, and mature blood cells, encompassing granulocytes, monocytes, dendritic cells, B and T lymphocytes, plasma cells, and pDCs (Figure 5D). These results provide direct evidence, at sc resolution, that adult mouse BM ECs can generate hematopoietic progenitors and mature blood cells.

Additionally, 30 “Mesenchymal type” cells (from cluster 14; co-expressing mesenchymal cell markers, *Runx1*, and the EC markers *Cdh5* and *Pecam1*) were also marked by 13 “true” barcodes, 9 of which were shared with hematopoietic cell progenitors (HSPC and Erythroid) and mature blood cells (Figure 5E and F). Also, 6 “true” barcodes linked “Mesenchymal type” cells to ECs (from clusters 0, 1, 22 and 13), suggesting either a shared precursor or derivation from each other. Although it cannot be excluded that barcoding missed identification of mesenchymal cell links to other ECs or cells, these results raise the possibility that certain BM “Mesenchymal type” cells may produce hematopoietic cell progeny. Despite similarities of sc tracing results linking ECs and “Mesenchymal type” cells to hematopoietic cells, these two cell populations display a distinctive transcriptome profile (Figure 5G; Figure S4E).

Additional analysis of all tracked cells showed that several ECs and to a lower extent “Mesenchymal type cells” shared “true” barcodes (Figure 5D–5F), indicating clonal expansion. Transcriptome-based sc cell cycle analysis, confirmed the presence of ECs and “Mesenchymal type cells” in the S and G2/M phases, albeit to a much lower degree than HSPC (Figure S5C). Together, these results demonstrate at a sc level that adult BM ECs can generate hematopoietic cell progeny of HSPC and mature blood cells. The results further raise the possibility that “Mesenchymal type” cells, marked by a hybrid endothelial and stromal phenotype, may represent an additional source of hemogenic activity in adult BM.

### Single cell transcriptome identifies a *Cdh5*^+^*Col1a2*^+^ *Runx1*^+^cell population in the adult BM.

**Figure 6.**
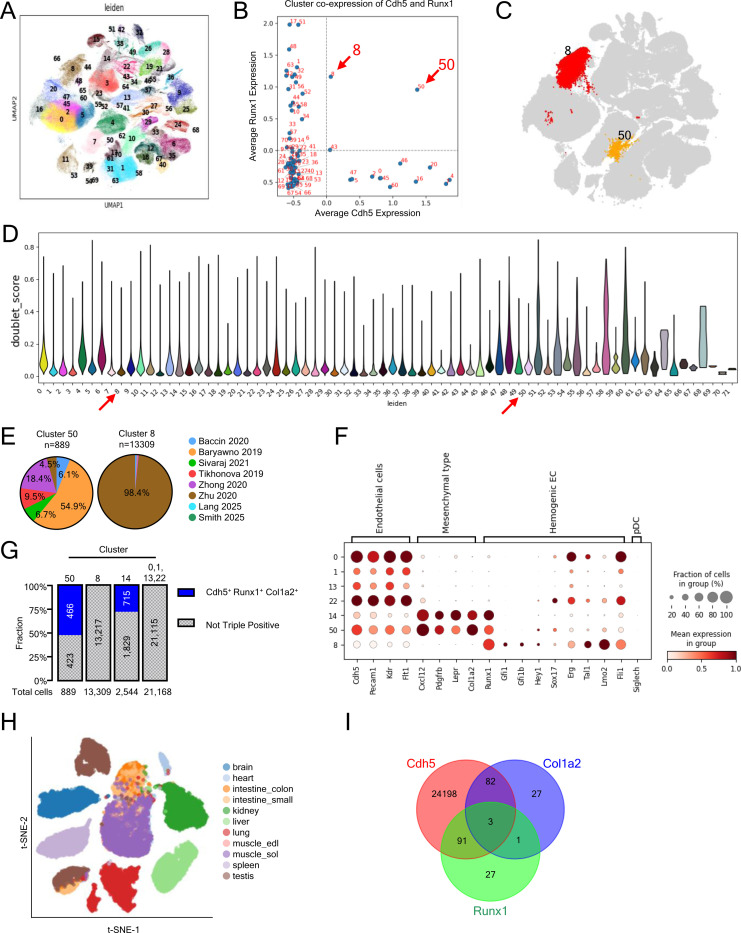
Sc transcriptomic analysis of prospective hemogenic ECs (A) UMAP clustering of 434,810 cells from eight public scRNA-seq datasets. (B) Dot plot showing relative Cdh5 and Runx1 co-expression across clusters; clusters 8 and 50 co-express both genes. (C) UMAP highlighting clusters 8 and 50; all other clusters shown in grey. (D) Violin plots of doublet scores across Leiden clusters. Clusters 50 and 8 show no evidence of doublet enrichment. (E) Datasets proportional contribution to clusters 50 and 8; each dataset is color-coded. (F) Dot plot showing expression of selected marker genes in clusters 50 and 8 (from the public sc RNA-seq datasets listed in Figure 7D) and from clusters 0, 1, 13, 22 and 14 (from Polylox scRNA-seq; Figure 5B). Results reflect mean expression and fraction of cells in group. (G) Cdh5, Runx1 and Col1a2 co-expression in the indicated clusters as a fraction of cells in the cluster. (H and I) t-SNE plot of ECs from 11 murine tissues (G) and Venn diagram (H) showing rare co-expression of Cdh5, Runx1, and Col1a2 in these tissues.

To further characterize adult hemogenic cell populations, we analyzed publicly available scRNAseq datasets comprising BM cells from adult mice (1–16 months of age)^53–59^ and embryonic caudal artery ECs (9.5–11.5 days post coitum)^24^. After quality control and dimensional reduction, the remaining 434,810 cells clustered into 71 distinct populations (Figure 6A-D). Among the *Cdh5*-expressing endothelial clusters, two clusters, cluster 8 composed predominantly of embryonic cells (98%) and cluster 50 composed largely of adult BM-derived cells (95.5%) (Figure 6E), were notable in comprising cells co-expressing *Cdh5* and *Runx1*, a transcription factor that marks the hemogenic EC identity during development^60^ (Figure 6B).

We jointly analyzed the transcriptome from the publicly available embryonic cluster 8 and adult cluster 50 (Figure 6 A-C), and from our sc Polylox dataset, including adult clusters 14 (Mesenchymal-type) and adult EC clusters 0, 1, 13 and 22 (Figure 5B). All populations expressed canonical EC markers (*Cdh5*, *Pecam1*, *Kdr*, *Flt1*), though at different levels, but expression of *Runx1* was mostly confined to clusters 8, 50 and Polylox 14 (Figure 6F). Interestingly, adult clusters 50 and Polylox 14 co-expressed the mesenchymal cell-associated genes *Col1a2*, *Lepr*, *Cxcl12*, and *Pdgfrb*, distinguishing these two clusters from the embryonic cluster 8 and adult Polylox clusters 0, 1, 13 and 22 (Figure 6F). Additionally, embryonic cluster 8 exhibited higher expression of key EHT-related transcription factors (FLI1, LMO2, TAL1, ERG)^61–64^, some of which were variably expressed by the Polylox clusters 0,1,13 and 22 (Figure 6F).

To explore whether cells expressing *Cdh5*, *Col1a2* and *Runx1* are unique to BM (Figure 6G), we looked at other adult mouse tissues. Analysis of public scRNAseq datasets from 11 adult mouse tissues^65^ showed that *Cdh5*, *Runx1*, and *Col1a2* expressing ECs are largely restricted to the BM, with only 3 such cells detected out of ~32,000 EC from other tissues (Figure 6H and I)^65^. These observations, together with the Polylox sc tracing experiments, indicate that adult mouse BM harbors a cell population with mixed endothelial and mesenchymal phenotype marked by *Runx1*, *Col1a2* and *Cdh5* expression and raises the possibility that this population has hemogenic potential.

### *Col1a2* and *Runx1* expression in BM ECs

To evaluate a potential contribution of *Col1a2* expression to EHT in adult BM, we generated the mouse lines Col1a2-Cre^ERT2^/mTmG and Col1a2-Cre^ERT2^/ZsGreen to track cells expressing the *Col1a2* gene (Figure S6A). Four weeks after tamoxifen administration, the BM of Col1a2-Cre^ERT2^/ZsGreen mice contained abundant ZsGreen^+^ cells (Figure S6B). By flow cytometry, a subset of these BM Col1a2-tracked ZsGreen^+^ cells were VE-Cadherin^+^CD45^−^ and RUNX1^+^ consistent with an endothelial identity and perhaps hemogenic potential (Figure S6C). Noteworthy, a similar subset of VE-Cadherin^+^CD45^-^RUNX1^+^ cells was detected in BM of adult WT C57Bl/6 mice (Figure S6D) and in BM of Cdh5-Cre^ERT2^(PAC)/ZsGreen mice after tamoxifen treatment (Figure S6E).

**Figure 7.**
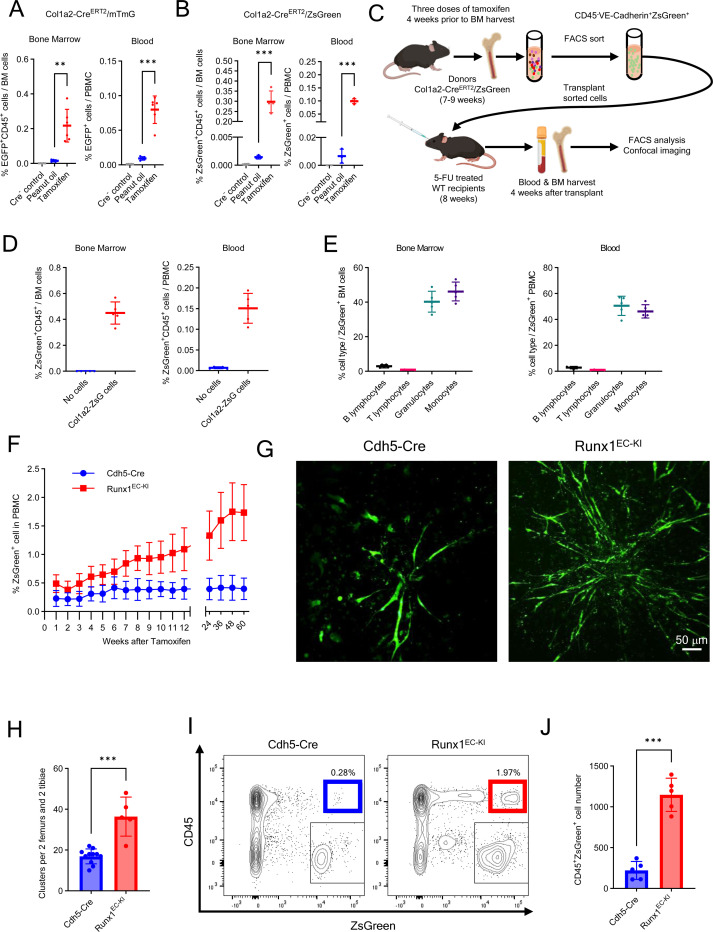
Contribution of *Col1a2* and *Runx1* expression to ECs hemogenic activity (A and B) Percent EGFP^+^CD45^+^ cells in BM and blood of tamoxifen-treated (n=6) or oil-treated (n=5) Col1a2-Cre^ERT2^/mTmG mice (A) and tamoxifen-treated (n=4) or oil-treated (n=3) Col1a2-Cre^ERT2^/ZsGreen mice (B). Cre-control mice (n=5 in A, and n=2 in B). (C) Transplant experiment: sorted VE-Cadherin^+^CD45^−^ZsGreen^+^/Col1a2^+^ cells from tamoxifen-treated Col1a2-Cre^ERT2^/ZsGreen mice are transplanted into 5-FU-conditioned WT recipients. (D and E) Detection (D) and characterization (E) of ZsGreen^+^CD45^+^ cells in BM and blood of WT 5-FU-conditioned mice (n=5), 4 weeks post-transplant of VE-Cadherin^+^CD45^−^ZsGreen^+^/Col1a2^+^ cells. Control FU-conditioned WT mice (n=4) received no cell transplant (D). (F) Time course of ZsGreen^+^ PBMC detection in control (Cdh5-Cre^+^/ZsGreen^+^) and Runx1^EC-KI^ (Cdh5-Cre^+^/ZsGreen^+^/Runx1-KI) mice (n=10 per group). (G and H) Representative images (G) and quantification (H) of ZsGreen^+^ cells from OP9 cell-supported cultures of BM cells from tamoxifen-treated Cdh5-Cre^+^/ZsGreen^+^ (n=11) and Runx1^EC-KI^ mice (n=5). (I and J) Representative flow cytometry plots (I) and quantification (J) of CD45^+^ZsGreen^+^ cells from OP9 cell-supported BM cell cultures (n=5/group). Dots represent individual mice. Data are shown as mean ± SD. **p<0.01, ***p < 0.001 by Student’s t test.

To evaluate hemogenic potential, we looked for Col1a2-tracked CD45^+^ hematopoietic cells in Col1a2-Cre^ERT2^/mTmG and Col1a2-Cre^ERT2^/ZsGreen mouse lines after tamoxifen treatment. In both these mouse lines, we detected CD45^+^ hematopoietic cells tracked by EGFP or ZsGreen fluorescence in BM and blood, which were rare but significantly more abundant than in control mice not treated with tamoxifen (Figure 7A and B; Figure S6F-H). These results indicated that a proportion of the Col1a2-tracked cells in the adult mouse are hemogenic.

To further evaluate this possibility, we first sorted VE-Cadherin^+^CD45^−^ ZsGreen (Col1a2)^+^ cells from the BM of tamoxifen-induced adult Col1a2-Cre^ERT2^/ZsGreen mice (Figure S7A), examined expression of selected genes, and used these cells in transplant experiments. The sorted VE-Cadherin^+^CD45^−^ ZsGreen (Col1a2)^+^ cells expressed *Cdh5*, *Col1a2*, *Cxcl12*, and *Runx1* mRNAs distinctively from other BM cell population (Figure S7B), but resembled the BM hemogenic population annotated as “mesenchymal-type” (cluster 14) identified by Polylox s.c sequencing (Figure 5G; Figure S4E) and subsets of adult BM Cdh5^+^ cells identified in public adult datasets (Figure 6E and F).

We transplanted the BM VE-Cadherin^+^CD45^−^ ZsGreen (Col1a2)^+^ cells (1×10^4^ cells/mouse) into 5-FU-conditioned adult WT C57Bl/6 recipients and looked for tracked CD45^+^ cells in the BM and blood (Figure 7C). Four weeks after transplantation, BM and peripheral blood of transplant recipients contained ZsGreen^+^CD45^+^ cells (Figure 7D; Figure S7C and D), indicating that the transplanted ZsGreen^+^ (Col1a2)-tracked CD45^−^ ECs had produced CD45^+^ hematopoietic cell progeny. These ZsGreen^+^CD45^+^ cells in the recipient mice comprised mainly granulocytes and monocytes, and few B and T lymphocytes (Figure 7E). These results indicate that Col1a2-tracked ECs, like Cdh5(VE-Cadherin)-tracked ECs, can give rise to hematopoietic progeny in vivo, but display a more restricted multilineage potential compared to ECs from Cdh5-Cre^ERT2^(PAC)/ZsGreen mice.

In additional experiments, we evaluated the role of *Runx1* expression in adult BM hemogenic EC since our analyses identified *Runx1* as a putative marker of adult EHT (Figure 6E and F; Figure S4E). Therefore, we generated a Runx1^EC-KI^ mouse line (*Cdh5-Cre^ERT2^/ZsGreen/Runx1-Knock-In*), in which Cre-mediated excision of a floxed STOP codon enables co-expression of ZsGreen and *Runx1* in ECs upon tamoxifen induction (Figure S7E)^66^. In these Runx1^EC-KI^ mice, tamoxifen treatment increased significantly the frequency of ZsGreen^+^ cells in PBMC over 60 weeks compared to control tamoxifen-treated (Cdh5-Cre^ERT2^/ZsGreen) mice (Figure 7F).

Since these circulating ZsGreen-tracked cells presumably represent hematopoietic cell progeny of ECs co-expressing ZsGreen and *Runx1*, we tested the hemogenic potential of these tamoxifen-induced BM EC ex vivo. Using the culture system that successfully supported ex vivo hematopoiesis in Cdh5-tracked BM (Figure 2A-H), we now compared the hemogenic potential of BM cells from tamoxifen pretreated Runx1^EC-KI^ mice to that of BM cells from tamoxifen pretreated Cdh5-Cre^ERT2^/ZsGreen) mice. We detected a significantly greater number of ZsGreen^+^ clusters in cultures from Runx1^EC-KI^ BM cells compared to control BM cells (Figure 7G and H), and flow cytometry showed that more CD45^+^ZsGreen^+^ hematopoietic cells were produced in cultures of Runx1^EC-KI^ BM cells compared to BM from controls (Figure 7I and J). Although we cannot exclude the possibility that RUNX1 promotes EC proliferation in culture, these results support a role of RUNX1 in promoting adult EHT.

## DISCUSSION

Our results provide evidence for the presence of ECs with hemogenic potential in the adult mouse BM. Previously, hemogenic ECs were detected during embryonic development or perinatally but not thereafter^1–9,67^. The current findings argue that EHT is not limited to the prenatal or perinatal development but is present up to 28 weeks after birth, decreasing thereafter. This conclusion is supported by Cdh5-based bulk and Polylox sc lineage tracking, culture of hemogenic ECs, transplant analyses and characterization of EC-derived hematopoietic cell progeny, which link key features of adult EHT to embryonic EHT^25–28,68^.

Previous experiments found that EHT, present in the late fetus/neonatal BM, disappears shortly after birth^67^. This contrasts with the current experiments showing persistence of adult EHT well beyond 20 weeks of age. The divergent results likely stem from functional differences of the Cdh5-based tracking systems. In the previous experiments, attempts to activate Cdh5-fluorescence in BM ECs by injecting tamoxifen at different time-points after birth were unsuccessful starting 20 days after birth. Expectedly, the absence of tamoxifen-induced fluorescence in BM ECs was associated with the absence of traced hematopoietic cell output from these cells. In the current experiments and those of others^36^, tamoxifen administration after 10, 20, and 30 weeks of age consistently induces fluorescence in most BM ECs. We conclude that the absence of adult EHT had not been firmly established.

Adult hemogenic ECs identified here express *Cdh5*, *Pecam1*, *Kdr*, and *Flt1*, resembling other BM EC populations and embryonic hemogenic ECs^69^. However, the current studies identify yet another, small hemogenic cell population in the adult BM, distinctive in co-expressing typical EC markers, *Cdh5* and *Pecam1*, and the mesenchymal-type markers *Lepr*, *Col1a2* and *Cxcl12*. These two hemogenic populations are clonally linked, but it is currently unclear whether they have a common progenitor, or one population derives from the other.

ECs derive from two sources during development: the splanchnic mesoderm that gives rise to the primitive aorta where ECs located on the aortic floor are hemogenic^68^, and the somites from the paraxial mesoderm, that produce ECs contributing the endothelial vascular network of the trunk and limb^70,71^. Somite-derived ECs are not hemogenic in situ, but they can transiently acquire a hemogenic potential when variously induced^72^ and may also include a cell subset with hemogenic potential^73^. They can also express *Runx1* and trigger aortic hematopoiesis from hemogenic ECs in zebrafish^74^ and in the chick^68^. Besides generating hemogenic ECs, blood and other tissues, the mesoderm in conjunction with the neural crest, gives rise to mesenchymal stem cells (MSCs) and perhaps a precursor of both MSCs and ECs^75–77^. However, MSCs do not produce blood cells, although they can differentiate into many other tissues^78^, and their potential for endothelial differentiation remains controversial^79–82^. By contrast, endothelial to mesenchymal transition (EndMT) is a well-established process in development and disease states^83^.

It was suggested that the transient wave of EHT occurring in the BM of the late fetus and perinatally may serve to mitigate the slow HSC colonization of BM from the fetal liver or perhaps prepare the BM niches to accommodate the incoming HSC from the liver, but a function has not yet been firmly established^67^. Similarly, the function of adult BM EHT identified here is currently unclear. We hypothesize that adult EHT plays a role under conditions of hematopoietic stress or disease rather than under steady-state conditions, but this will need future investigation. Interestingly, previous studies found that subsets of EC can regenerate after irradiation that has eliminated HSC^45^, and we traced the emergence of some hematopoietic cells from ECs that persisted after lethal mouse irradiation. We also found that 5-FU treatment was required for the successful engraftment and function of adult hemogenic ECs suggesting that this population may require inductive signals from a BM that is recovering from an insult^45,84^. The identification of such inductive factors may enable effective propagation of BM hemogenic ECs ex vivo and motivate a search for hemogenic ECs in human BM.

Altogether, our results provide evidence for a previously unrecognized capacity of ECs in the adult mouse BM to generate blood cells. These results suggest that hematopoiesis in the adult mouse may arise through the contribution of cells and processes beyond the HSCs generated through aortic EHT during development and BM EHT perinatally.

### Limitations of the study

A limitation of our study is that the function of adult EHT is currently unknown. Under steady-state conditions, the hematopoietic cell output from adult EHT is low in comparison to that of embryonic EHT, which effectively sustains adult hematopoiesis, and our experiments detected no functional differences between hematopoietic cells from embryonic and adult EHT. To address this limitation, we will examine whether adult EHT is a greater contributor to adult hematopoiesis when stress is imposed on the adult hematopoietic system, and more fully characterize the cell populations arising from adult EHT, focusing on minor cell population known to serve critical functions in specific settings. Another limitation of our study is that we could not extend the in vivo single-cell PolyloxExpress genetic lineage tracing to the ex vivo single-cell genetic lineage tracing due to technical limitations of the PolyloxExpress system. Finally, rigorous serial transplantation experiments, including CD45.1/CD45.2 competitive repopulation assays, will be necessary to conclusively determine whether bona fide long-term hematopoietic stem cells are generated by adult hemogenic endothelial cells both *in vivo* and *ex vivo*.

## RESOURCE AVAILABILITY

### Lead contact

Further information and requests for resources and reagents should be directed to and will be fulfilled by the lead contact, Giovanna Tosato (tosatog@mail.nih.gov).

### Materials availability

This study did not generate new unique reagents.

### Data and code availability

The results of scRNAseq and PacBio SmrtSeq of Polylox barcodes are deposited in NCBI SRA (PRJNA1079369, public at time of publication). A custom script was used to retrieve cell indexes from the PolyloxExpress amplicons (https://github.com/CCRSF-IFX/SF_Polylox-BC). Bash, R, and Python codes are available from the corresponding author upon reasonable request.

## METHODS

### EXPERIMENTAL MODEL AND STUDY PARTICIPANT DETAILS

#### Mouse strains

All animal studies were approved by the Institutional Animal Care and Use Committee of the CCR (Bethesda, MD), National Cancer Institute (NCI), NIH and conducted in adherence to the NIH Guide for the Care and Use of Laboratory Animals (National Academies Press, 2011) and approved protocols. *Cdh5-Cre^ERT2^(PAC)* mice ^37^ (MGI:3848982) were a gift from Dr. R. Adams and *Cdh5-Cre^ERT2^(BAC)* mice ^39^ (MGI:5705396) were a gift from Dr. Kubota. *Col1a2-CreER* mice ^85^ were purchased from the Jackson Laboratory (JAX#029567). Cre-dependent *Ai6-ZsGreen* ^86^ (JAX#007906) and *mTmG* ^87^ (JAX#007676) fluorescent reporter mice were purchased from the Jackson Laboratory. In *ZsGreen* reporter mice, Cre activity leads to constitutive expression of ZsGreen1 in cell bodies. In *mTmG* reporter mice, Cre activity leads to an irreversible switch from cell membrane-localized tdTomato (mT) to membrane-localized EGFP (mG). *PolyloxExpress* mice ^33^ were a kind gift of Drs. Hans-Reimer Rodewald and Avinash Bhandoola. The Cdh5-Cre^ERT2^/ZsGreen/*PolyloxExpress* mouse line was generated in house. Runx1 conditional knock-in mice^66^ (Gt(ROSA)26Sor^tm1(CAG-Runx1)Lzjg^**,** MGI:7490340) were generously provided by Drs. Qiufu Ma and Nancy Speck. Runx1 endothelial-specific knock-in (*Runx1^EC-KI^*) mice were generated by crossing *Runx1^Ki/+^* mice with *Cdh5-Cre^ERT2^(PAC)/ZsGreen^Tg/Tg^* mice. Upon tamoxifen treatment, Cre-mediated excision of the two floxed STOP codons enables co-expression of Runx1 and ZsGreen in ECs.

All animals were bred in the animal facilities of CCR/NCI (Bethesda, MD). The mice were maintained in a C57BL/6J background. Mice were identified with ear tags and routinely genotyped by PCR. No mouse was excluded from the experiments, unless assessed as sick by the veterinarians or fight wounds were observed at harvest. Tamoxifen (Sigma-Aldrich, #T5648) dissolved in peanut oil (Sigma-Aldrich, #P2144) (10 mg∙mL^−1^) was administered orally (via gavage using 22g feeding needles) at 100 mg∙kg^−1^. Unless otherwise specified, three doses on consecutive days were administered. Unless indicated otherwise, 8- to 12-week-old male and female mice were used when tamoxifen was administrated. No randomization or blinding was used to allocate experimental groups. Mice were typically sacrificed between 9 AM and 11 AM local time.

### METHOD DETAILS

#### OP9 cell culture

OP9 cells, a gift from Dr. T. Nakano^44^ (also available from ATCC, #CRL-2749), were maintained in α-MEM (Gibco #12561056; without ribonucleosides and deoxyribonucleosides, with 2.2 g/L sodium bicarbonate) supplemented with 20% fetal bovine serum (FBS; Sigma-Aldrich #2442). Culture dishes and flasks (Corning #353003, #430641U) were precoated with gelatin (Sigma-Aldrich #G9391; ~100 μL/cm², 60 minutes at 37°C). Cells were incubated at 37°C in a humidified atmosphere of 95% air and 5% CO_2_.

#### Bone marrow cell harvesting, culture and terminal harvest for analysis

To harvest BM cells to be cultured, mice were euthanized by cervical dislocation and soaked in 70% ethanol for 5 minutes. Long bones (femurs and tibiae) were dissected, and surrounding skin, muscle, and connective tissue were carefully removed. Cleaned bones were immediately transferred into ice-cold sterile PBS and kept on ice. Once all bones were harvested, they were soaked in 70% ethanol for 1 minute and rinsed three times with ice-cold PBS.

Each bone was cut into two pieces using sterile scissors and placed--with the open end facing downward-- into a sterile 500 μL microcentrifuge tube pre-perforated with a 16G needle (sterilized in advance). Each 500 μL tube was loaded with two femurs and two tibiae. To each tube, 200 μL of DMEM supplemented with 1 mM EDTA was added. The 500 μL tubes were then placed inside sterile 1.5 mL microcentrifuge tubes, sealed with Parafilm, and centrifuged at 12,000 rpm for 20 seconds. The inner tubes were discarded, and the BM pellet collected in the 1.5 mL tubes was resuspended in 1 mL DMEM + 1 mM EDTA.

Cells were filtered through 70 μm cell strainers, centrifuged at 350 × g for 5 minutes at 4°C, and resuspended in complete DMEM (DMEM containing 15% FBS (Sigma-Aldrich #2442–500ML, LOT:24G002), Penicillin-Streptomycin [Gibco #15140–122], and Anti-Anti [Gibco #15240–062]). A single-cell suspension was prepared by pipetting 30 times with a 1 mL pipette. Bone marrow cells were cultured either on Corning Primaria^™^ dishes/flasks (Corning #353808, #353810, #353846) or on OP9 stromal cell monolayers in standard tissue culture dishes/flasks (Corning #353003, #430641U) precoated with gelatin (Sigma-Aldrich #G9391; ~100 μL/cm², incubated 60 min at 37°C).

Cells were seeded at a density equivalent to BM cells from two femurs and two tibiae per ~75 cm² culture surface (e.g., T75 flask) in 15 mL complete DMEM. After ~32 hours, non-adherent cells and medium were removed, and adherent cells were gently washed three times with PBS. For Primaria cultures, 15 mL fresh complete DMEM was replenished twice weekly. For OP9 co-cultures, non-adherent cells and medium were removed twice weekly and replaced with fresh complete DMEM supplemented with freshly isolated unfractionated WT BM cells (from two femurs and two tibiae per ~75 cm² surface, using the same BM isolation method mentioned above).

For terminal collection, at the start of the final culture week, all non-adherent cells were removed, and adherent cells were washed three times with PBS. Fresh complete DMEM (15 mL) was added. After three days, a second 15 mL complete DMEM addition was made. No further medium changes occurred before harvest. For final collection, non-adherent cells and supernatant were collected first. Adherent cells were washed with PBS, and the wash was pooled with the supernatant. Remaining adherent cells (including OP9 and BM-derived cells) were detached using 0.25% Trypsin-EDTA (Gibco #25200–056) for 5 minutes at 37°C and added to the pooled suspension. After another PBS wash, a second trypsinization (10 minutes at 37°C) was performed. All collected material from each step was combined for downstream flow cytometric analysis. For transplantation experiments, only non-adherent cells (with supernatant) and loosely adherent cells recovered after the initial 5-minute Trypsin-EDTA incubation were pooled and subjected to FACS sorting to isolate ZsGreen^+^ cells for injection.

#### Blood collection

For terminal collection, blood was obtained from the mouse abdominal aorta with BD Vacutainer^™^ EDTA tubes (BD #367856) and Vaculet^™^ blood collection needles (23G, EXELINT #26766). Blood smears were prepared with 10 μL of collected blood. For flow cytometry analysis, ACK buffer (Lonza, #BP10–548E) was added to the blood to lyse red blood cells before Fc receptor blocking and antibody staining. For non-terminal blood collection, ~20–50μL blood was collected by submandibular blood sampling, using a 3mm animal lancet (BRAINTREE SCI., GR-3MM) and a 250μL BD Microtainer^®^ K2EDTA tube (BD #365974). Kwik Stop^®^ Styptic Powder was applied to stop the bleeding (Miracle Corp. #423615). For long-term, repeated blood collection, 2 drops of blood were collected from the tail vein with Microhematocrit Capillary Tubes (Fisherbrand # 22–362574). White blood cell (WBC) counts were determined using acridine orange/propidium iodide (AO/PI, Logos Biosystems, #F23001) staining and quantified with a LUNA-FL^™^ fluorescence cell counter (Logos Biosystems). For flow cytometry detection of ZsGreen/EGFP positive PBMCs, blood was collected with one of the methods above. For flow cytometric detection of ZsGreen^+^/EGFP^+^ PBMCs, blood was collected using one of the three methods described above. Red blood cells were lysed with ACK buffer, and DAPI and DRAQ5 were used to exclude dead cells and to identify nucleated PBMCs.

#### Bone marrow harvest for flow cytometry analysis

For flow cytometry analysis, bone marrow was harvested using one of the two methods described below. For hematopoietic cell isolation, bone marrow was harvested by flushing femurs and tibiae with ice-cold Sort Buffer (1× PBS [Gibco, #10010–031] supplemented with 5 mM EDTA, 25 mM HEPES, and 2% FBS [Sigma-Aldrich, #F2442]). Red blood cells were then lysed using ACK lysing buffer (Lonza, #10–548E) according to the manufacturer’s instructions. Cells were then washed with Sort Buffer and passed through a 40μm cell strainer (GREINER BIO-ONE, #542040, #542140). For greater preservation of endothelial cells, bone marrows were harvested by gently crushing mouse femurs and tibiae in Sort Buffer (5mM EDTA, 25mM HEPES, 2% FBS in 1× PBS). Red cell lysis was performed using ACK buffer. Bone marrow cells were then incubated with 0.1U∙Ml^−1^ Collagenase (Worthington Biomedical Corp., #LS004176), 0.8U∙mL^−1^ Dispase (Worthington Biomedical Corp., #LS02109), and 0.5mg∙mL^−1^ DNase (Worthington Biomedical Corp., #LS006344) in 1x Hanks’ Balanced Salt Solution (HBSS) with Ca^2+^ and Mg^2+^ (Gibco, # 14065056) at 37°C for 30 min on a rotating mixer. Cells were then washed with Sort Buffer and passed through a 40μm cell strainer.

#### Endothelial cell transplantation experiments

Four days prior to transplant, recipient mice received one dose of 5-FU (150 mg∙kg^−1^ in PBS, Sigma-Aldrich, #F6627) intraperitoneally under isoflurane anesthesia. Sorted bone marrow cells (Col1a2-Cre/ZsGreen: 5,000 cells; Cdh5-Cre/ZsGreen: 20,000 cells in 100μL PBS) were inoculated retro-orbitally under isoflurane anesthesia. Bone marrows and blood were harvested from transplant recipients four weeks after the transplant unless otherwise specified.

#### Bone marrow ablation and transplantation

Recipient mice were lethally irradiated (11 Gy) using a Cesium-137 (^137^Cs) gamma irradiator three days prior to transplantation.

For whole BM cell transplantation into *mTmG* recipients, BM cells were harvested from WT donor mice by flushing (as described above); 5 × 10^6^ cells were transplanted per recipient via tail vein.

For LSK transplantation into WT recipients, BM from Cdh5-Cre^ERT2^(PAC)/ZsGreen mice (not treated with tamoxifen) was harvested by flushing, followed by red blood cell lysis with ACK buffer. Fc receptor blocking was performed using Azide-Free Fc Receptor Blocker (Innovex, #NB335–60) per manufacturer’s instructions. Cells were stained with antibodies to Lineage cocktail, Sca-1, and c-Kit. DAPI was used to exclude dead cells. LSK populations were sorted using Sony SH800S and its Ultra Purity mode. Transplanted cell numbers were as follows: ZsGreen^−^ LSKs (5 × 10^4^, n = 3; 2.5 × 10^4^, n = 3) and ZsGreen^+^-enriched LSKs (2,800 cells, n = 2).

For transplantation of ex vivo–cultured BM cells, non-adherent and loosely adherent ZsGreen^+^ cells were collected from 8-week OP9 co-cultures (as described above), sorted by FACS for live (DAPI^-^) ZsGreen^+^ cells, and transplanted into irradiated recipients at 5 × 10^4^, 2.5 × 10^4^, or 1.25 × 10^4^ cells per mouse (n = 2 per group).

#### Flow cytometry and cell sorting

For intracellular antigen detection, single cell suspensions of bone marrow and blood were first incubated with Azide-Free Fc Receptor Blocker (Innovex, #NB335–60), following the manufacturer’s instructions. After washing, cells were first stained with surface marker antibodies at the concentration of 2μg per 1×10^7^ cells in Sort Buffer for 30 minutes at 4°C and then stained with live/dead cell discriminating BioLegend Zombie Dyes (UV, NIR, Violet, or Yellow, BioLegend #423108, #423106, #423114, and # 423104) following the manufacturer’s instructions. After washing, cells were fixed in 4% paraformaldehyde for 10 minutes at 37°C, and then permeabilized with 1% saponin (Sigma-Aldrich, # 47036) /Sort Buffer for 30min on ice. Subsequently, the cells were stained with rat monoclonal primary RUNX1-PE Ab (Invitrogen, # 12–9816-80) in 0.1% saponin/Sort Buffer overnight. After washing and resuspension, propidium iodide (PI, 0.5μM, Millipore Sigma, # P4170), 7-AAD (1μg∙mL^−1^, Millipore Sigma, #A9400) or DAPI (0.5μg∙mL^−1^, BioLegend, #422801) was added as a nuclear counterstain. For live cell staining without cell permeabilization, after cell surface antibody staining, cells were washed and suspended in Sort buffer containing propidium iodide (PI, 0.5μM, Millipore Sigma, # P4170), 7-AAD (1μg∙mL^−1^, Millipore Sigma, #A9400) or DAPI (0.5μg∙mL^−1^, BioLegend, # 422801), to distinguish dead cells from the live cells. For FACS sorting of live endothelial cells, after Fc receptor blocking, bone marrow cells were first depleted of CD45^+^ cells with MojoSort mouse CD45 nanobeads (BioLegend #480028) following the manufacturer’s protocol and then stained with specific antibodies. For flow cytometry analysis, compensation beads (BD Biosciences, #552844) were used for flow cytometer compensation. Flow cytometric data were acquired with BD FACSCanto-II, BD LSRFortessa, BD FACSymphony A5 (BD Biosciences), Sony SA3800 or Sony ID7000 cell analyzers. Cell sorting was performed with BD FACS Aria III, BD FACS Aria Fusion or Sony SH800S cell sorters. FSC and SSC profiles were used for excluding dead cells and debris. 7-AAD, PI, DAPI or BioLegend Zombie Dye was used for excluding dead cells. FSC-W versus FSC-H and SSC-W versus SSC-H were used to gate on single cells. Unless otherwise specified, fluorescence minus one (FMO) controls are used for negative gating reference. For BM HSPC analysis, BM cells were harvested followed by lineage positive cell depletion (Biolegend #480004). Data were analyzed with FlowJo (BD, v10.8.1), SONY ID7000 Software (Version 1.2.0.28212) or FACS Diva (BD, v6.1 and v9.0). Flowjo Plugins UMAP_R (v4.0.4) and FlowSOM (v4.1.0) were used for UMAP dimensional reduction and unsupervised clustering of flow cytometry data.

#### Bone marrow cryosections

Deeply anesthetized mice were transcardially perfused with 20mL ice-cold 1x PBS, followed by perfusion with 15mL ice-cold hydrogel solution (5% acrylamide/bis-acrylamide 19:1 (Sigma-Aldrich, #A2917), 2.5mg/mL polymerization initiator VA-044 (FUJIFILM, Wako, VA-044, Water soluble Azo initiators), 4% PFA in 1× PBS, 5mL/min flow speed). Femurs and tibiae were collected in tubes containing 5mL hydrogel solution and incubated at 4°C for 4 hours. The bones were then washed with PBS and incubated at 37°C for 2 hours. Bone decalcification was performed by incubating the bones in 40mL 0.5M EDTA pH 8.0 (KD Medical, #RGF-3130) for 3 days on a rotate mixer, with daily refreshed 0.5M EDTA solution. The bones were then dehydrated in 20% sucrose and 2% polyvinylpyrrolidone in PBS overnight. Dehydrated bones were then embedded in OCT (SAKURA, #4583) blocks using Precision Cryoembedding System (IHC WORLD, #IW-P101). Cryosections (10μm) for immunofluorescence staining were prepared from OCT frozen bone blocks using Leica CM3050S microtome, low-profile microtome blades (Leica 819, #14035838925), and TruBond^™^ 380 adhesion slides (Electron Microscopy Sciences, # 63701-W10).

#### Immunofluorescence staining and imaging

Tissue sections were rehydrated with 1× PBS (15 minutes), permeabilized in 0.3% Triton X-100 (Sigma-Aldrich, #T9284)/PBS (15 minutes), washed in 1× PBS, and incubated (2 hours) with blocking solution (2% BSA, 5% donkey serum (SIGMA, #D9663), and 0.3% Triton X-100/PBS). Samples were then washed 3 times with PBS and incubated with primary antibodies (5ng/mL; 4°C overnight). When secondary antibodies were used, 3 PBS washes were performed before incubating with fluorescent-labeled secondary antibodies (2ng/mL, room temperature, 2 hours). After washing (3x, 10 minutes each with 1× PBS), DAPI was added (300nM in PBS, 10 min). After 3 washes (5 min each with 1× PBS), coverslips were mounted (EPREDIA, #9990402), dried and sealed with nail polish. For blood smear staining, slides were first soaked in acetone/methanol/PFA (19:19:2 for 90 seconds) before rehydration ^88^. Confocal imaging was performed with Zeiss LSM 780, Zeiss LSM 880 NLO Two Photon, or Nikon *ECLIPSE* Ti2-E SoRa systems, according to the experimental specific needs (resolution, speed, wavelength capabilities). Images were processed with Zen (Zen Black v2.3, release Version 14.0.12.201, Zen Blue Lite v2.5, Carl Zeiss), Bitplane Imaris (v9.7.0, Oxford instruments), and Photoshop (v23.3.0, Adobe, for whole image contrast and brightness adjustments).

#### Isolation of peritoneal cavity cells

To isolate peritoneal cavity cells, mice were euthanized by cervical dislocation, injected intraperitoneally (i.p.) with cold FACS sort buffer (5 mL), massaged and flicked gently on the abdomen. Peritoneal fluid was then withdrawn slowly and transferred into a polypropylene centrifuge tube on ice prior to centrifugation (350×g, 10min, at 4°C) and analysis.

#### Thioglycolate induced sterile peritonitis

Mice were injected i.p. with 1 mL PBS or 4% thioglycolate (Sigma-Aldrich, #70157). Peritoneal cavity cells were harvested 4 hours after injection for further analysis.

#### Phagocytosis assay

Phagocytosis assay was performed using pHrodo^™^ Red *Escherichia coli* BioParticles (Invitrogen, # P35361) following the manufacturer’s instructions. Briefly, mice were first treated with thioglycolate (as described). Peritoneal cavity cells were incubated with the fluorescent *Escherichia coli* particles for 60 min at 37°C. Cells were then stained with surface marker antibodies and analyzed by flow cytometry.

#### ROS assay

ROS assay was performed using CellROX^™^ Orange Flow Cytometry Assay Kit (Invitrogen, # C10493) following the manufacturer’s instructions. Briefly, mice were first treated with thioglycolate (as described). CellROX detection reagent was added to peritoneal cavity cells (final concentration of 500nM) prior to incubation for 60 min at 37°C. Cells were then stained with the surface marker antibodies and analyzed by flow cytometry.

#### PolyloxExpress single cell lineage tracing

These experiments essentially followed published procedures ^33^. Briefly, BM cells from tamoxifen treated Cdh5-Cre/ZsGreen/Polylox mice (treated at 10 weeks old and harvested at 16 weeks old) were enriched for ZsGreen positive cells by FACS. Single cell capturing was performed with 10x Genomics Chromium Single Cell 3′ Reagent Kits, following manufacturer’s protocols. After reverse transcription (RT) in droplets, pooled cDNA was amplified and split into two aliquots for parallel transcriptome library preparation and barcode enrichment. Initial library quality control was performed with Agilent TapeStation D5000. For transcriptome analysis, 10 μL (25%) of a 10x cDNA library was fragmented and a gene expression library, generated following protocols in Single Cell 3’ Reagent Kits v3 and v4, was sequenced with Illumina NextSeq 2000 P3/P4 Reagents (28+74 bp read length). For Polylox barcode amplification, targeted amplification of barcodes from a 5–10 ng aliquot of a 10x cDNA library was performed by nested PCR. In the first round, primers #2,652 (5′-GCATGGACGAGCTGTACAAG-3′, annealing at the 5′ end of Polylox) and #2,674 (5′-AATGATACGGCGACCACCGAGATCTACACTCTTTCCCTACACGACGCTC-3′, annealing at the adaptor site (read 1) were used for amplification for 5 min at 95°C; (30 s at 95°C, 30 s at 57°C, 3 min at 72°C) 12 times; 10 min at 72°C. PCR products purified with 0.7x AMPure beads were used for the second round of PCR using primers #2,426 (5′-CGACGACACTGCCAAAGATTTC-3′, annealing at the 5′ end of Polylox) and #2,676 (5′-AATGATACGGCGACCACCGA-3′, annealing at the 5′ end of primer #2,674), for 5 min at 95°C; (30 s at 95°C, 30 s at 60°C, 3 min at 72°C) 18 times; 10 min at 72°C. The PCR products were purified with AMPure PB beads according to the manufacturer’s protocol. Long read amplicon-seq libraries were sequenced by PacBio Sequel II with PacBio Amplicons Library Preparation using SMRTbell prep kit 3.0. A custom transcriptome reference was built from mouse reference MM10 to include ZsGreen1.

A snakemake workflow with custom python script was used to retrieve cell indexes and Polylox barcodes from the PolyloxExpress amplicons (https://github.com/CCRSF-IFX/SF_Polylox-BC). Single cell transcriptome and single cell barcodes were linked using the 10x 3’ kit cell index and group information. Further analysis and illustrations were generated using Scanpy (https://github.com/scverse/scanpy). Doublet detection was performed using Scrublet, with the predicted doublet rate calculated based on 10x Genomics guidance about the cell numbers loaded to the microfluidic chips. Batch correction was done using BBKNN method (https://github.com/Teichlab/bbknn). Cell types were manually annotated based on canonical marker gene expression, guided by results from three automated annotation tools: (1) decoupleR ((https://saezlab.github.io/decoupleR/), using PangLaoDB (https://panglaodb.se/) as reference; (2) scANVI (https://github.com/scverse/scvi-tools) using ImmGne (https://www.immgen.org/) as reference; (3) CellTypist, using the embedded Immune_All_Low model. The rare barcodes and their pGen were identified using the MatLab script from the Höefer’s Lab (https://github.com/hoefer-lab/polylox). True barcodes are defined as pGen < 1×10^−4^, such that the expectation of a True barcode in detected 4,072 cells is 0.407. This work utilized the computational resources of the NIH HPC Biowulf cluster (http://hpc.nih.gov) and Frederick Research Computing Environment (FRCE). Python version: 3.10.; R version 4.5.0.

#### Public single-cell RNA-seq data analysis

Raw FASTQ and BAM files were downloaded from publicly available datasets (GSE108885 ^54^, GSE108891 ^54^, GSE118436 ^54^, GSE123078 ^54^, GSE122465 ^55^, GSE128423 ^53^, GSE145477 ^65^, GSE156635 ^56^, GSE137116 ^24^, E-MTAB-8077 ^65^, GSE230260^58^, GSE259382^59^). The BAM files were first converted to FASTQ files with bamtofastq (10x Genomics, v2.0.1). All FASTQ files were then processed by the count function of Cell Ranger (10x Genomics, v8.0.1) and aligned to the mouse genome (mm10, version 2020-A), to generate read matrixes. Further analysis and illustrations were generated using Scanpy as described above.

#### Definition of cultured fluorescent BM cell clusters and quantification

A cluster was defined as (1) a spatially distinct group of ZsGreen^+^ cells not contiguous with other fluorescent cells, (2) containing a central core with at least five extending branches, and (3) exhibiting a roughly radial organization, with projections spreading outward in multiple directions, and (4) extending at least ~200 μm in one direction. Isolated or irregularly scattered fluorescent cells were not counted. Due to their large size, clusters were counted manually across the culture dish using a tally counter under fluorescence microscopy.

#### RNA isolation and quantitative RT PCR

RNA was extracted using RNeasy^®^ Micro Kit (QIAGEN, #74004), following the manufacturer’s protocol. Cells were sorted directly into RNA lysis buffer (Buffer RTL of RNeasy^®^ Micro Kit). cDNA samples were prepared with SuperScript IV Reverse Transcriptase (Invitrogen, #18091050), following manufacturer's instructions. Real Time PCR was performed using Applied Biosystems^™^ TaqMan^™^ Fast Advanced Master Mix (4444557) and Applied Biosystem^™^ QuantStudio^™^ 5 Real-Time PCR System. TaqMan^™^ probes used were purchased from Applied Biosystems^™^: Spp1 (Mm00436767_m1); Cxcl12 (Mm00445553_m1); Col1a2 (Mm00483888_m1); Cdh5 (Mm00486938_m1); Runx1 (Mm01213404_m1); Ptprc (Mm01293577_m1); Gapdh (Mm99999915_g1); Actb (Mm02619580_g1). The reaction condition was set as follows: 50°C 2 minutes, 95°C 20 seconds, 45 cycles of 95°C 1 second, 60°C 20 seconds. Ct values were determined using the ABI QuantStudio^™^ Design & Analysis Software (v1.5.2). Relative gene expression was assessed using the 2^−ΔΔCt^ method, normalized to Gapdh expression level for each sample. The data was further normalized to gene expression levels in the unsorted bone marrow sample to calculate relative gene expression levels in each sample. Data reflect triplicates real-time PRC experiments.

### QUANTIFICATION AND STATISTICAL ANALYSIS

No statistical method was used to predetermine sample size. No data were excluded from the analyses. Mice with the correct genotypes were randomly assigned to control or treated groups. Unless otherwise specified, data are represented as mean ± S.D, and individual dots in the graphs indicate individual mice. Comparisons between two groups were performed using two-tailed unpaired Student’s *t*-tests (except for Figure S1-H, which used a paired *t*-test). Spearman rank correlation test was used for Figure 3E. Statistical analyses were performed with GraphPad Prism (v9.0.1). A statistical difference of *P*<0.05 was considered significant: ns, not significant, * *P* < 0.05, ** *P* < 0.01, *** *P*< 0.001.

## Supplementary Material

Supplement 1

## Figures and Tables

**Table:** RESOURCES TABLE

REAGENT or RESOURCE	SOURCE	IDENTIFIER
Antibodies		
BD Horizon^™^ BV786 Rat Anti-Mouse CD117	BD Biosciences	564012; RRID:AB_2732005
Brilliant Violet 421^™^ anti-mouse CD117 (c-Kit) Antibody	BioLegend	105828; RRID:AB_11204256
Brilliant Violet 785^™^ anti-mouse CD117 (c-Kit) Antibody	BioLegend	105841; RRID:AB_2629799
BD Pharmingen^™^ APC Rat Anti-CD11b	BD Biosciences	553312; RRID:AB_398535
PerCP/Cyanine5.5 anti-mouse CD150 (SLAM) Antibody	BioLegend	115922; RRID:AB_2303663
APC/Fire^™^ 750 anti-mouse CD150 (SLAM) Antibody	BioLegend	115940; RRID:AB_2629587
BD Pharmingen^™^ APC-Cy^™^7 Rat Anti-Mouse CD19	BD Biosciences	557655; RRID:AB_396770
BD Horizon^™^ BUV737 Rat Anti-Mouse CD19	BD Biosciences	612781; RRID:AB_2870110
Brilliant Violet 510^™^ anti-mouse CD3 Antibody	BioLegend	100234; RRID:AB_2562555
BD Pharmingen^™^ Purified Rat Anti-Mouse CD31	BD Biosciences	553370; RRID:AB_394816
BV421 anti-mouse Cd31	BD Biosciences	563356; RRID:AB_2738154
Brilliant Violet 421^™^ anti-mouse CD31 Antibody	BioLegend	102424; RRID:AB_2650892
Brilliant Violet 605^™^ anti-mouse CD31 Antibody	BioLegend	102427; RRID:AB_2563982
BD Pharmingen^™^ Alexa Fluor^®^ 700 Rat Anti-Mouse CD45	BD Biosciences	560510; RRID:AB_1645208
BD OptiBuild^™^ BUV615 Rat Anti-Mouse CD45	BD Biosciences	751170; RRID:AB_2875194
PE anti-mouse CD45 Antibody	BioLegend	103106; RRID:AB_312971
PerCP/Cyanine5.5 anti-mouse CD45 Antibody	BioLegend	103131; RRID:AB_893344 103132; RRID:AB_893340
BD Horizon^™^ BV510 Hamster Anti-Mouse CD48	BD Biosciences	563536; RRID:AB_2738266
APC/Cyanine7 anti-mouse CD48 Antibody	BioLegend	103432; RRID:AB_2561463
Brilliant Violet 510^™^ anti-mouse CD48 Antibody	BioLegend	103443; RRID:AB_2650826
Collagen I Polyclonal Antibody, Biotin	Invitrogen	PA1–28530; RRID: AB_1956957
Endomucin Monoclonal Antibody (eBioV.7C7 (V.7C7)), eFluor^™^ 660	Invitrogen	50–5851-82; RRID:AB_11220465
Anti-Endomucin Antibody (V.7C7) AF546	Santa Cruz Biotechnology	sc-65495 AF546; RRID:AB_2100037
BD Pharmingen^™^ PE-Cy^™^7 Rat Anti-Mouse Ly-6G	BD Biosciences	560601; RRID:AB_1727562
BD Horizon^™^ BUV395 Rat Anti-Mouse Ly-6G	BD Biosciences	563978; RRID:AB_2716852
BD Pharmingen^™^ APC Mouse Lineage Antibody Cocktail, with Isotype Control	BD Biosciences	558074; RRID:AB_1645213
Goat anti-Rat IgG (H+L) Highly Cross-Adsorbed Secondary Antibody, Alexa Fluor^™^ Plus 594	Invitrogen	A48264; RRID:AB_2896333
RUNX1 Monoclonal Antibody (RXDMC), PE, eBioscience^™^	Invitrogen	12–9816-80; RRID:AB_11151519
BD Pharmingen^™^ PE-Cy^™^7 Rat Anti-Mouse Ly-6A/E	BD Biosciences	561021; RRID:AB_2034021
PE/Cy7 anti-mouse Ly-6A/E (Sca-1)	BioLegend	108114; RRID:AB_493596
PerCP/Cyanine5.5 anti-mouse TER-119/Erythroid Cells Antibody	BioLegend	116228; RRID:AB_893636
Brilliant Violet 605^™^ anti-mouse TER-119/Erythroid Cells Antibody	BioLegend	116239; RRID:AB_2562447
BD Pharmingen^™^ Alexa Fluor^®^ 647 Rat Anti-Mouse CD144	BD Biosciences	562242; RRID:AB_2737608
BD Pharmingen^™^ PE Rat Anti-Mouse CD144	BD Biosciences	562243; RRID:AB_2737609
BUV737 Rat Anti-Mouse CD144	BD Biosciences	741792; RRID:AB_2871138
Alexa Fluor^®^ 647 anti-mouse CD144 (VE-cadherin) Antibody	BioLegend	138006; RRID:AB_10569114
BD OptiBuild^™^ RB780 Rat Anti-Mouse CD144	BD Biosciences	755945; RRID:AB_3683567
PE anti-mouse CD144 (VE-cadherin) Antibody	BioLegend	138010; RRID:AB_10641139
PE/Cyanine7 anti-mouse CD144 (VE-cadherin) Antibody	BioLegend	138015; RRID:AB_2562885 138016; RRID:AB_2562886
PE anti-mouse CD144 (VE-cadherin) Antibody	BioLegend	138105; RRID:AB_2077941
**Chemicals, peptides, and recombinant proteins**		
5-FLUOROURACIL	Sigma-Aldrich	F6627
7-AAD Viability Stain SOLN	Life Technologies Corp.	00–6993-50
7-Aminoactinomycin D	Sigma-Aldrich	A9400
ACETONE	MALLINCKRODT	2440
ACK lysing Buffer	Lonza	10–548E
Acrylamide/Bis 19:1, 40% (w/v) solution	Invitrogen	AM9024
Acrylamide/Bis-acrylamide 19:1	Sigma-Aldrich	A2917
Acridine Orange / Propidium Iodide Stain	Logos Biosystems	F23001
AMPure beads	Beckman Coulter	A63881
Antibiotic-Antimycotic	Gibco	15240062
Anti-Rat Ig, κ/Negative Control (BSA) Compensation Plus (7.5 μm) Particles Set	BD Biosciences	560499
Anti-Rat Ig, κ/Negative Control Compensation Particles Set	BD Biosciences	552844
APC/Fire^™^ 750 Streptavidin	BioLegend	405250
Azide-Free Fc Receptor Blocker	INNOVEX	NB335–60
Bovine Serum Albumin solution	MP Biomedicals	160069
CellROX Orange Flow Cytometry Assay Kit	Invitrogen	C10493
Collagenase Type 2	Worthington Biochemical Corporation	LS004176
Dispase	Worthington Biomedical	LS02109
Deoxyribonuclease	Worthington Biomedical	LS006344
DAPI (4',6-Diamidino-2-Phenylindole, Dilactate)	BioLegend	422801
Dihydroethidium (Hydroethidine)	Invitrogen	D1168
Donkey serum	Sigma-Aldrich	D9663
DRAQ5	Biostatus	DR50200
EDTA (0.5M, pH 8.0)	KD Medical	RGF3130
ETHYL ALCOHOL (200 PROOF ANHYDROUS)	Warner-Graham Co.	201096
Ethylenediamine-N,N,N′,N′-tetra-2-propanol	Sigma-Aldrich	8219401000
Fetal Bovine Serum	Millipore Sigma	F2442–500ML (Lot 24G002)
Fc Receptor Blocker	Innovex	NB309–30
Formalin solution, neutral buffered, 10%	Sigma-Aldrich	HT501128–4L
Gelatin	Sigma-Aldrich	G9391
Goldenrod^™^ Animal Lancet	Braintree Scientific Inc.	GR-3MM
HBSS	Gibco	14025075
HEPES	Gibco	15630080
Immu-Mount^™^ mountant	Epredia	9990402
Isoflurane	Baxter	10019036040
Mag-Bind^®^ TotalPure NGS	Omega Bio-tek	M1378–01
Methanol, HPLC Grade	Avantor	JT-9093–03
MojoSort^™^ Mouse CD45 Nanobeads	BioLegend	480028
Kwik Stop^®^ Stypic Power	Miracle Care	SKU 423615
Lineage Cell Depletion Kit, mouse	MiltenyiBiotec	130–090-858
Lipopolysaccharides	Sigma Aldrich	L2880–25MG
Microscope Slides	MATSUNAMI GLASS IND.	SUMGP12
Neutral Protease, Partially Purified, Animal Free/AF	Worthington Biochemical Corporation	LS02109
Paraformaldehyde (formaldehyde) aqueous solution (20%)	Electron Microscopy Sciences	15713-S
Penicillin-Streptomycin	Gibco	15140–122
Peanut oil	Sigma-Aldrich	P2144
Phusion Green Hot Start II High-Fidelity PCR Master Mix	Thermo Scientific	F566L
pHrodo^™^ Red E. coli BioParticles	Invitrogen	P35361
Polyvinylpyrrolidone	Sigma-Aldrich	P5288
Propidium iodide	Sigma-Aldrich	P4170
Q5 Hot Start High-Fidelity 2X Master Mix	NEB	M0494S
Richard-Allan Scientific^™^ Cover glass	Epredia	102424
RNeasy^®^ Micro Kit	QIAGEN	74004
Saponin	Sigma-Aldrich	47036
SPRIselect Beads	Beckman Coulter	B23318
Sucrose	Sigma-Aldrich	S8501
SuperScript^™^ IV First-Strand Synthesis System	Invitrogen	18091050
Tamoxifen	Sigma-Aldrich	T5648
TaqMan^™^ Fast Advanced Master Mix	ThermoFisher Scientific	4444557
Thioglycollate Broth	Sigma Aldrich	70157
Tissue-Tek^®^ O.C.T. Compound	SAKURA	4583
Triton^™^ X-100	Sigma-Aldrich	T9284
Trypsin-EDTA (0.25%), phenol red	Gibco	25200056
TruBond^™^ 380 Adhesion Slide	Electron Microscopy Sciences	63700-Y10
UNI-TRIEVE	INNOVEX	NB325
VA-044 (Water soluble Azo initiators)	FUJIFILM Labchem Wako	VA-044
VECTASHIELD Vibrance Antifade Mounting Medium	Vector Laboratories	H-1700–10
Zombie Aqua^™^ Fixable Viability Kit	BioLegend	423101
Zombie NIR^™^ Fixable Viability Kit	BioLegend	423105
Zombie UV^™^ Fixable Viability Kit	BioLegend	423107
Zombie Yellow^™^ Fixable Viability Kit	BioLegend	423103
Zymosan A from Saccharomyces cerevisiae	Sigma Aldrich	Z4250
**Critical commercial assays**		
Chromium GEM-X Single Cell 3' Kit	10× Genomics	1000686
Chromium GEM-X Single Cell 3' Chip Kit v4	10× Genomics	1000690
Chromium Next GEM Chip G Single Cell Kit	10× Genomics	1000127
Chromium Next GEM Single Cell 3' Kit v3.1	10× Genomics	1000269
Dual Index Kit TT Set	10× Genomics	1000215
NextSeq 2000 P4 Reagents (100 Cycles)	Illumina	20100994
NextSeq 2000 P3 Reagents (100 Cycles)	Illumina	20040559
**Deposited data**		
Polylox PacBio long-read sequencing data	This Study	PRJNA1079369
Single cell RNA seq data	This Study	PRJNA1079369
**Experimental models: Cell lines**		
OP9 cells	Dr. Nakano; same line deposited in ATCC	ATCC # CRL-2749; RRID:CVCL_4398
**Experimental models: Organisms/strains**		
*Cdh5-Cre(PAC)^ERT2^*	Drs. R. Adams and M. Boehm; also available from Taconic Biosciences	MGI:3848982 Taconic # 13073
*Cdh5-Cre(BAC)^ERT2^*	Drs. Y. Kubota and Y. Mukoyama	MGI:5705396
*Col1a2-CreERT*	JAX, #029567	MGI:3785760
*ZsGreen (Ai6)*	JAX, #007906	MGI:3809522
*mTmG*	JAX, #007676	MGI:3716464
*PolyloxExpress*	Drs. H. Rodewald and A. Bhandoola	MGI:6470648
*Runx1Knock-In*	Drs. Q. Ma and N. Speck	MGI:7490340
**Oligonucleotides**		
*mTmG* mouse strain genotyping primers (5’ – 3’)	CTT TAA GCC TGC CCA GAA GA TAG AGC TTG CGG AAC CCT TC AGG GAG CTG CAG TGG AGT AG	JAX: 007676
*ZsGreen* mouse strain genotyping primers (5’ – 3’)	AAG GGA GCT GCA GTG GAG TA CCG AAA ATC TGT GGG AAG TC GGC ATT AAA GCA GCG TAT CC AAC CAG AAG TGG CAC CTG AC	JAX: 007906
*Cdh5-Cre^ERT2^(PAC)* mouse strain genotyping primers (5’ – 3’)	TCC TGA TGG TGC CTA TCC TC CCT GTT TTG CAC GTT CAC CG CAC CCT GTT CTT TGC CTC CT	This study.
*Cdh5-Cre^ERT2^(BAC)* mouse strain genotyping primers (5’ – 3’)	ATA CCG GAG ATC ATG CAA GC ATG TGA ACC AGC TCC CTG TC CTA GGC CAC AGA ATT GAA AGA TCT GTA GGT GGA AAT TCT AGC ATC ATC C	JAX: Protocol 029211
*Col1a2-Cre^ERT^* mouse strain genotyping primers (5’ – 3’)	CAT GTC CAT CAG GTT CTT GC TGA AAA AGT CCA CTA ATT AAA ACC A CTA ACA ACC CTT TCT CTC AAG GT CAG GAG GTT TCG ACT AAG TTG G	JAX: Protocol 19893
*Runx1 Knock-In* mouse strain genotyping primers (5’ – 3’)	GAG TTC TCT GCT GCC TCC TGG CGA GGG CAG CCA TAG CAA CTC CGA GGC GGA TCA CAA GCA ATA	Qi, et al., 2017
*PolyloxExpress* mouse strain genotyping primers (5’ – 3’)	AAG GGA GCT GCA GTG GAG TA TAA GCC TGC CCA GAA GAC TCC AAG ACC GCG AAG AGT TTG TCC	Pei, et al., 2020
**Software and algorithms**		
FlowJo v10	BD	https://www.flowjo.com RRID:SCR_008520
GraphPad Prism 10	Dotmatics	www.graphpad.com RRID:SCR_002798
Snakemake	Köster et al.	https://snakemake.github.io/ RRID:SCR_003475
Lima	PacBio	https://lima.how/ RRID:SCR_025520
Iso-Seq	PacBio	https://isoseq.how/ RRID:SCR_025481
Minimap2	Li, et al (2021)	https://github.com/lh3/minimap2 RRID:SCR_018550
Cell Ranger	10x Genomics	V8.0.1, RRID:SCR_017344
Polylox barcode recovery	This study	https://github.com/CCRSF-IFX/SF_Polylox-BC
pGen calculation	Pei, et al. (2020)	https://github.com/hoefer-lab/polylox
Scanpy	Scanpy Community	https://scanpy.readthedocs.io RRID:SCR_018139
rapids-singlecell	Rapids-SingleCell	https://rapids-singlecell.readthedocs.io
decoupler	Badia-i-Mompel et al. (2022)	https://decoupler-py.readthedocs.io
scANVI	Gayoso et al. (2022)	https://github.com/scverse/scvi-tools
CellTypist	Xu et al. (2021)	https://celltypist.readthedocs.io/
**Other**		
Primaria^™^ dishes/flasks	Corning	353808; 353810; 353846
Regular Cell Culture Flask	Corning	430641U
