# Supplementary material for: Single-cell lineage tracing identifies hemogenic endothelial cells in the adult mouse bone marrow
Source: bioRxiv. 2025 Oct 10:2025.10.09.681472. Preprint. [Version 1] doi: 10.1101/2025.10.09.681472 (PMC12747260; doi:10.1101/2025.10.09.681472)
Supplement: Supplement 1 [file media-1.pdf]

Figure S1

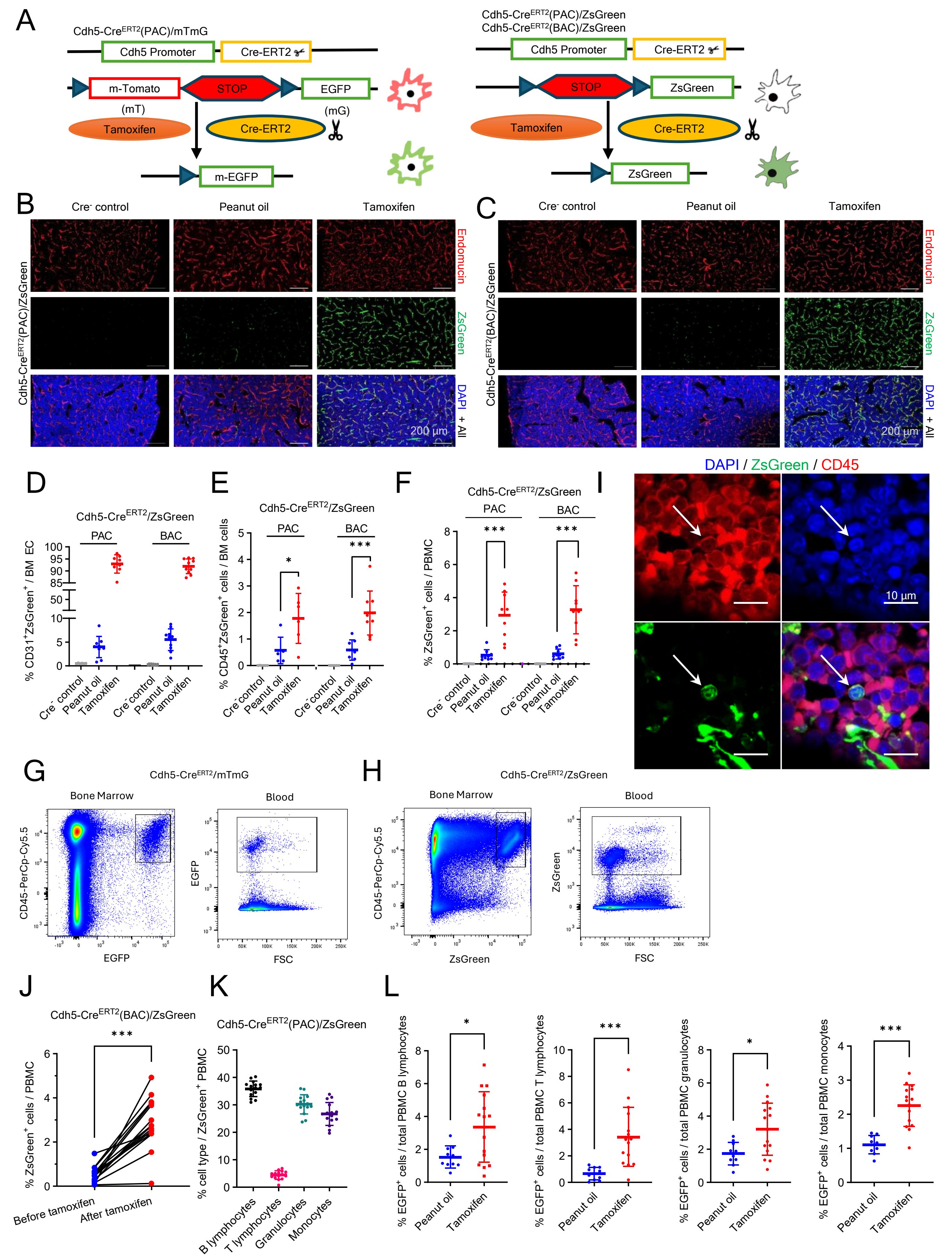
A
Cdh5-CreERT2(PAC)/mTmG
Cdh5 Promoter
Cre-ERT2
m-Tomato
STOP
EGFP
(mT)
(mG)
Tamoxifen
Cre-ERT2
m-EGFP
Cdh5-CreERT2(PAC)/ZsGreen
Cdh5-CreERT2(BAC)/ZsGreen
ZsGreen
B
Cre- control
Peanut oil
Tamoxifen
Cdh5-CreERT2(PAC)/ZsGreen
Endomucin
ZsGreen
DAPI + All
200 µm
C
Cdh5-CreERT2(BAC)/ZsGreen
D
Cdh5-CreERT2/ZsGreen
PAC
BAC
% CD31+ZsGreen+ / BM EC
E
% CD45+ZsGreen+ cells / BM cells
F
% ZsGreen+ cells / PBMC
I
DAPI / ZsGreen / CD45
10 µm
G
Cdh5-CreERT2/mTmG
Bone Marrow
Blood
CD45-PerCp-Cy5.5
EGFP
FSC
H
Cdh5-CreERT2/ZsGreen
ZsGreen
J
Cdh5-CreERT2(BAC)/ZsGreen
% ZsGreen+ cells / PBMC
Before tamoxifen
After tamoxifen
K
Cdh5-CreERT2(PAC)/ZsGreen
% cell type / ZsGreen+ PBMC
B lymphocytes
T lymphocytes
Granulocytes
Monocytes
L
% EGFP+ cells / total PBMC B lymphocytes
% EGFP+ cells / total PBMC T lymphocytes
% EGFP+ cells / total PBMC granulocytes
% EGFP+ cells / total PBMC monocytes

Figure S1. Contribution of ECs to hematopoiesis in adult BM is revealed by Cdh5-Cre^{ERT2} mouse tracking lines. Related to Figure 1.

(A) Cdh5-tracking mouse lines. Tamoxifen switches-on green fluorescence in cells that express the Cre-recombinase and their cell progeny.

(B and C) Confocal microscopy images of representative BM sections from Cdh5-Cre^{ERT2}(PAC)/ZsGreen (B) and Cdh5-Cre^{ERT2}(BAC)/ZsGreen (C) adult mice showing tamoxifen-induced ZsGreen fluorescence co-staining of most Endomucin^{+} cells. Control BM sections from representative Cre^{+} mouse treated with peanut oil (no tamoxifen) display occasional ZsGreen^{+}Endomucin^{+} cells but no tamoxifen-independent ZsGreen fluorescence is detected in representative Cre^{-} mice.

(D) Flow cytometry analysis of adult BM cells from Cdh5-Cre^{ERT2}(PAC)/ZsGreen (n = 6-8) and Cdh5-Cre^{ERT2}(BAC)/ZsGreen mice (n = 10) shows that ZsGreen fluorescence identifies most ECs four weeks after tamoxifen administration but also tracks a small proportion of EC expressing tamoxifen-independent fluorescence in Cre^{+} but not Cre^{-} mice.

(E) Percent CD45^{+}ZsGreen^{+} cells of viable BM cells from Cre^{-} control (n=6), Cre^{+} control (peanut-oil treated, no tamoxifen; n=8) and Cre^{+} tamoxifen-treated Cdh5-Cre^{ERT2}(PAC) /ZsGreen (n=6) or Cdh5-Cre^{ERT2}(BAC)/ZsGreen mice (n=9). Mice were 8- to 12-week-old at the time of tamoxifen administration.

(F) Percent ZsGreen^{+} cells of PBMC from Cre^{-} control, Cre^{+} control (peanut-oil treated) and Cre^{+} tamoxifen-treated Cdh5-Cre^{ERT2}(PAC)/ZsGreen and Cdh5-Cre^{ERT2}(BAC)/ZsGreen mice (n=9/group). Mice were 8- to 12-week-old at the time of tamoxifen administration.

(G and H) Representative flow cytometry gating for CD45^{+}EGFP^{+} cells from bone marrow and blood of Cdh5-CreERT2/mTmG mice (G, relates to Figure 1C and D), and CD45^{+}ZsGreen^{+} cells from bone marrow and blood of Cdh5-Cre^{ERT2}/ZsGreen mice (H, relates to Figure S1E and F).

(I) Representative confocal images showing a nucleated (DAPI^{+}) BM ZsGreen^{+}CD45^{+} cell in the BM from a Cdh5-Cre^{ERT2}(PAC)/ZsGreen mouse treated with tamoxifen.

(J) Percent ZsGreen^{+} cells of PBMC in individual Cdh5-Cre^{ERT2}(BAC)/ZsGreen mice before or four weeks after tamoxifen administration. Each dot represents the results from 50-250µl blood/mouse. The lines link results from individual mice (n=15, 8- to 12-week-old at the time of tamoxifen administration).

(K) Percent B and T-lymphocytes, granulocytes, and monocytes among EGFP^{+} PBMC of Cre^{+} tamoxifen-treated Cdh5-Cre^{ERT2}(PAC)/mTmG mice (n=15, 8- to 12-week-old at the time of tamoxifen administration).

(L) Percent EGFP^{+} cells in peripheral blood cell populations of Cre^{+} peanut oil-treated (n=9) and tamoxifen-treated (n=16) Cdh5-Cre^{ERT2}(PAC)/mTmG mice (8- to 12-weeks-old at the time of tamoxifen administration). Dots represent individual mice. Data are shown as mean ± SD. *$p<0.05$, **$p<0.01$, ***$p < 0.001$ by Student's t test.

Figure S2

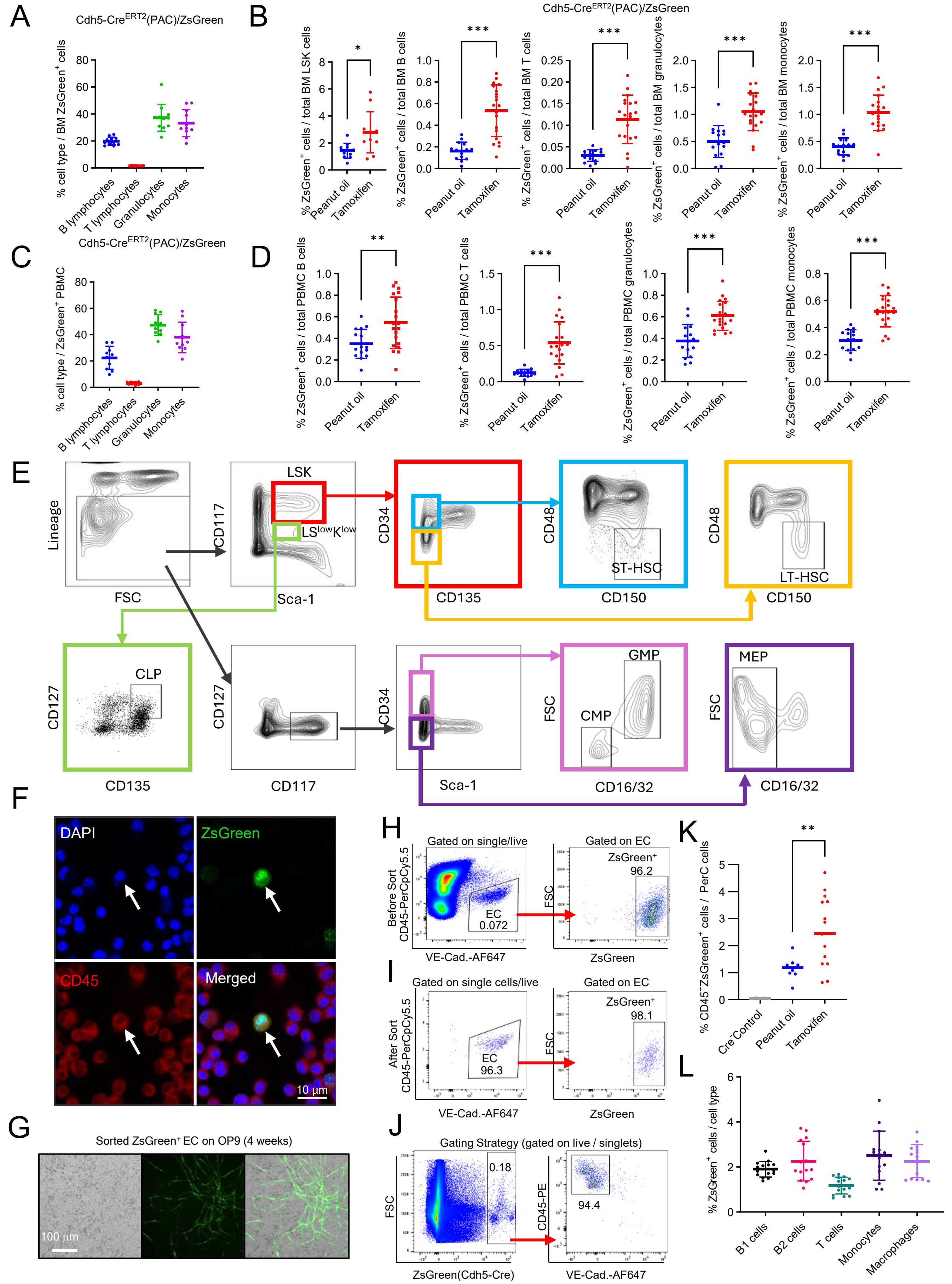

A
Cdh5-CreERT2(PAC)/ZsGreen
% cell type / BM ZsGreen+ cells
B lymphocytes
T lymphocytes
Granulocytes
Monocytes
B
Cdh5-CreERT2(PAC)/ZsGreen
% ZsGreen+ cells / total BM LSK cells
% ZsGreen+ cells / total BM B cells
% ZsGreen+ cells / total BM T cells
% ZsGreen+ cells / total BM granulocytes
% ZsGreen+ cells / total BM monocytes
Peanut oil
Tamoxifen
C
Cdh5-CreERT2(PAC)/ZsGreen
% cell type / ZsGreen+ PBMC
D
% ZsGreen+ cells / total PBMC B cells
% ZsGreen+ cells / total PBMC T cells
% ZsGreen+ cells / total PBMC granulocytes
% ZsGreen+ cells / total PBMC monocytes
E
Lineage
FSC
LSK
CD117
Sca-1
LSlowKlow
CD34
CD135
CD48
CD150
ST-HSC
LT-HSC
CLP
CD127
CMP
GMP
MEP
CD16/32
F
DAPI
ZsGreen
CD45
Merged
10 µm
G
Sorted ZsGreen+ EC on OP9 (4 weeks)
100 µm
H
Gated on single/live
Gated on EC
Before Sort CD45-PerCpCy5.5
EC 0.072
ZsGreen+ 96.2
VE-Cad.-AF647
ZsGreen
I
Gated on single cells/live
Gated on EC
After Sort CD45-PerCpCy5.5
EC 96.3
ZsGreen+ 98.1
J
Gating Strategy (gated on live / singlets)
0.18
94.4
ZsGreen(Cdh5-Cre)
CD45-PE
K
% CD45+ZsGreeen+ cells / PerC cells
Cre-Control
Peanut oil
Tamoxifen
L
% ZsGreen+ cells / cell type
B1 cells
B2 cells
T cells
Monocytes
Macrophages

Figure S2. Characterization of tracked hematopoietic progenitors and mature cells in adult BM and peripheral blood of Cdh5-Cre reporter mice. Related to Figure 1, 2, or 3.

(A and B) Percent B lymphocytes, T lymphocytes, granulocytes, and monocytes of all $ZsGreen^+$ cells (A) and percent $ZsGreen^+$ cells of total BM LSK, B and T-lymphocytes, granulocytes, and monocytes (B). Each dot reflects results from individual mice (1 femur plus 1 tibia combined, n=11); group means ± SD (error bars) are shown by the horizontal lines.

(C and D) Percent B lymphocytes, T lymphocytes, granulocytes, and monocytes of all $ZsGreen^+$ PBMC in Cdh5-Cre^{ERT2}(PAC)/ZsGreen mice treated with tamoxifen (C, n=10) and percent $ZsGreen^+$ cells of peripheral blood B lymphocytes, T lymphocytes, granulocytes, and monocytes in Cdh5-Cre^{ERT2}(PAC)/ZsGreen mice (D) treated with peanut oil (n=15) or tamoxifen (n=20).

(E) Gating strategy for identification of HSPC subsets in bone marrow. Representative flow cytometry plots show sequential gating of lineage negative (Lin^-) $Sca1^+$ $cKit^+$ (LSK) cells into long-term hematopoietic stem cells (LT-HSC), short-term hematopoietic stem cells (ST-HSC), multipotent progenitors (MPP), common lymphoid progenitors (CLP), common myeloid progenitors (CMP), megakaryocyte-erythroid progenitors (MEP), and granulocyte-macrophage progenitors (GMP).

(F) Representative cytospin image of floating and low-adherent cells from ex vivo culture of BM cells from a tamoxifen-induced Cdh5-Cre^{ERT2}(PAC)/ZsGreen mouse.

(G) Representative image showing the morphology of sorted BM $ZsGreen^+$ ECs from a tamoxifen-induced Cdh5-Cre^{ERT2}(PAC)/ZsGreen mouse after 4-week culture on OP9 cells.

(H) Gating strategy for sorting VE-$Cadherin^+$ $ZsGreen^+$ ECs from the BM of Cdh5-Cre^{ERT2}(PAC)/ZsGreen mice.

(I) Purity analysis of sorted VE-$Cadherin^+$ $ZsGreen^+$ ECs.

(J) Gating strategy used for detecting $ZsGreen^+CD45^+$ hematopoietic cells in WT C57Bl/6 recipients of ECs sorted from the BM of Cdh5-Cre^{ERT2}(PAC)/ZsGreen donors.

(K) Percent $CD45^+ZsGreen^+$ cells recovered from the peritoneal cavity (PerC) of Cre^+Cdh5-Cre^{ERT2}/ZsGreen mice treated with peanut oil (n=8) or tamoxifen (n=15). Cre^- mice (n=5).

(L) Percent $ZsGreen^+$ cells within PerC cell populations recovered from mice (n=15) under steady-state conditions. Cell type identification; B1 cells: $CD19^+$, $CD3^-$, $CD45R(B220)^-$, $CD5^+$, $CD43^+$; B2 cells: $CD19^+$, $CD3^-$, $CD45R(B220)^+$, $CD5^-$, $CD43^-$; T cells: $CD3^+$, $CD11b^-$; monocytes: $CD11b^+$, $CD19^-$, $Ly6G^-$, $Ly6C^{high}$; neutrophils: $CD11b^+$, $CD19^-$, $Ly6G^+$, $Ly6C^{low}$; and macrophages: $CD11b^+$, $CD19^-$, $F4/80^+$.

Dots represent individual mice. Data are shown as mean ± SD. *p<0.05, **p<0.01, ***p < 0.001 by Student's t test.

Figure S3

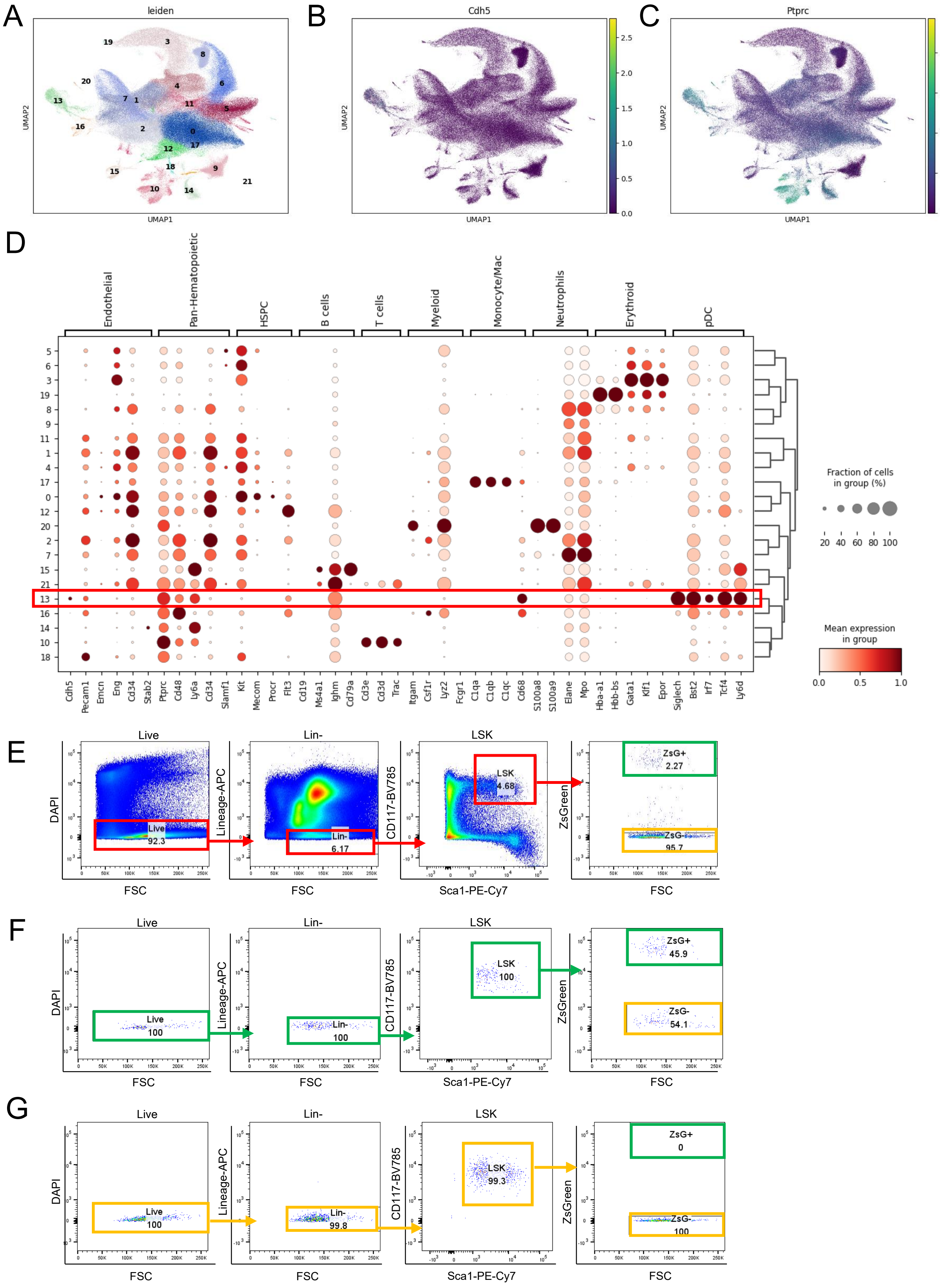
A
leiden
UMAP1
UMAP2
B
Cdh5
C
Ptprc
D
Endothelial
Pan-Hematopoietic
HSPC
B cells
T cells
Myeloid
Monocyte/Mac
Neutrophils
Erythroid
pDC
Cdh5 Pecam1 Emcn Eng Cd34 Stab2 Ptprc Cd48 Ly6a Cd34 Slamf1 Kit Mecom Procr Flt3 Cd19 Ms4a1 Ighm Cd79a Cd3e Cd3d Trac Itgam Csf1r Lyz2 Fcgr1 C1qa C1qb C1qc Cd68 S100a8 S100a9 Elane Mpo Hba-a1 Hbb-bs Gata1 Klf1 Epor Siglech Bst2 Irf7 Tcf4 Ly6d
5 6 3 19 8 9 11 1 4 17 0 12 20 2 7 15 21 13 16 14 10 18
Fraction of cells in group (%)
20 40 60 80 100
Mean expression in group
0.0 0.5 1.0
E
Live
Lin-
LSK
DAPI
Lineage-APC
CD117-BV785
ZsGreen
FSC
Sca1-PE-Cy7
Live 92.3
Lin- 6.17
LSK 4.68
ZsG+ 2.27
ZsG- 95.7
F
Live 100
Lin- 100
LSK 100
ZsG+ 45.9
ZsG- 54.1
G
Live 100
Lin- 99.8
LSK 99.3
ZsG+ 0
ZsG- 100

Figure S3 BM pDCs, but not other BM hematopoietic cells, express *Cdh5* and *Ptprc*, encoding CD45. Related to Figure 4.
(A) UMAP plot showing unsupervised clustering of sc transcriptomic data from a public dataset of mouse BM hematopoietic cells.
(B and C) UMAP plots displaying expression of *Cdh5* (B) and *Ptprc* (encoding CD45, C) in the dataset shown in (A).
(D) Dot plot illustrating the expression of selected marker genes across clusters shown in (A). The red rectangle highlights gene expression by cluster 13 cells, identifying pDCs. Dot size represents the percentage of cells expressing the gene within each cluster, and color intensity reflects the mean expression level.
(E) Gating strategy for selecting $ZsGreen^-$ and $ZsGreen^+$ LSK progenitors.
(F and G) Analysis of purity of sorted LSK populations enriched for $ZsGreen^+$ cells (F) and depleted of $ZsGreen^+$ cells (G) from the BM of Cdh5-Cre^{ERT2}(PAC)/ZsGreen mice (not treated with tamoxifen).

Figure S4

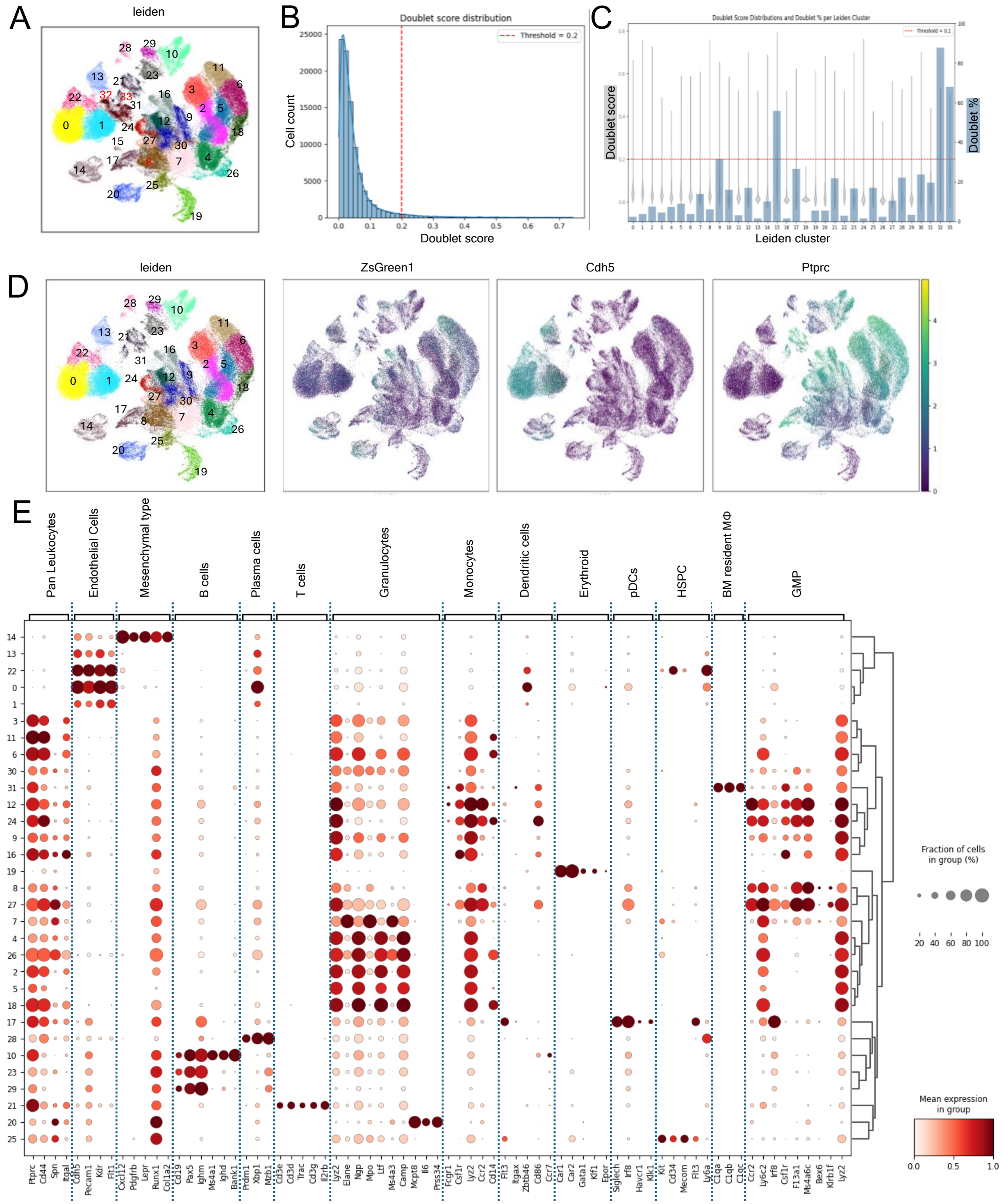

A
leiden
B
Doublet score distribution
Threshold = 0.2
Cell count
Doublet score
C
Doublet Score Distributions and Doublet % per Leiden Cluster
Doublet score
Doublet %
Leiden cluster
D
leiden
ZsGreen1
Cdh5
Ptprc
E
Pan Leukocytes
Endothelial Cells
Mesenchymal type
B cells
Plasma cells
T cells
Granulocytes
Monocytes
Dendritic cells
Erythroid
pDCs
HSPC
BM resident MΦ
GMP
Ptprc
Cd44
Spn
Itgal
Cdh5
Pecam1
Kdr
Flt1
Cxcl12
Pdgfrb
Lepr
Runx1
Col1a2
Cd19
Pax5
Ighm
Ms4a1
Ighd
Bank1
Prdm1
Xbp1
Mzb1
Cd3e
Cd3d
Trac
Cd3g
Il2rb
Lyz2
Elane
Ngp
Mpo
Ltf
Ms4a3
Camp
Mcpt8
Il6
Prss34
Fcgr1
Csf1r
Lyz2
Ccr2
Cd14
Flt3
Itgax
Zbtb46
Cd86
Ccr7
Car1
Car2
Gata1
Klf1
Epor
Siglech
Irf8
Havcr1
Klk1
Kit
Cd34
Mecom
Flt3
Ly6a
C1qa
C1qb
C1qc
Ccr2
Ly6c2
Irf8
Csf1r
F13a1
Ms4a6c
Bex6
Klrb1f
Lyz2
Fraction of cells in group (%)
20 40 60 80 100
Mean expression in group
0.0 0.5 1.0

Figure S4. Single-cell RNA-seq analysis of BM ZsGreen^{+} cells from tamoxifen-treated Cdh5-Cre/ZsGreen/Polylox mice. Related to Figure 5.

(A) UMAP plot showing unsupervised clustering of sc RNA-seq data, identifying 34 distinct cell clusters within BM ZsGreen^{+} cells.

(B) Histogram of doublet score distribution. A threshold of 0.2 was applied to match the expected doublet rate from 10x Genomics Chromium GEM-X chips.

(C) Doublet score distribution (gray violin plots, left Y-axis) and corresponding doublet percentages (blue bars, right Y-axis) across Leiden clusters.

(D) UMAP plots of clusters after doublet removal, and expression of *ZsGreen1*, *Cdh5*, and *Ptprc* (CD45).

(E) Dot plot showing expression of selected marker genes across Leiden clusters identified in (A). Dot size indicates the proportion of cells expressing the gene; color intensity reflects the average expression level of each cluster.

Figure S5

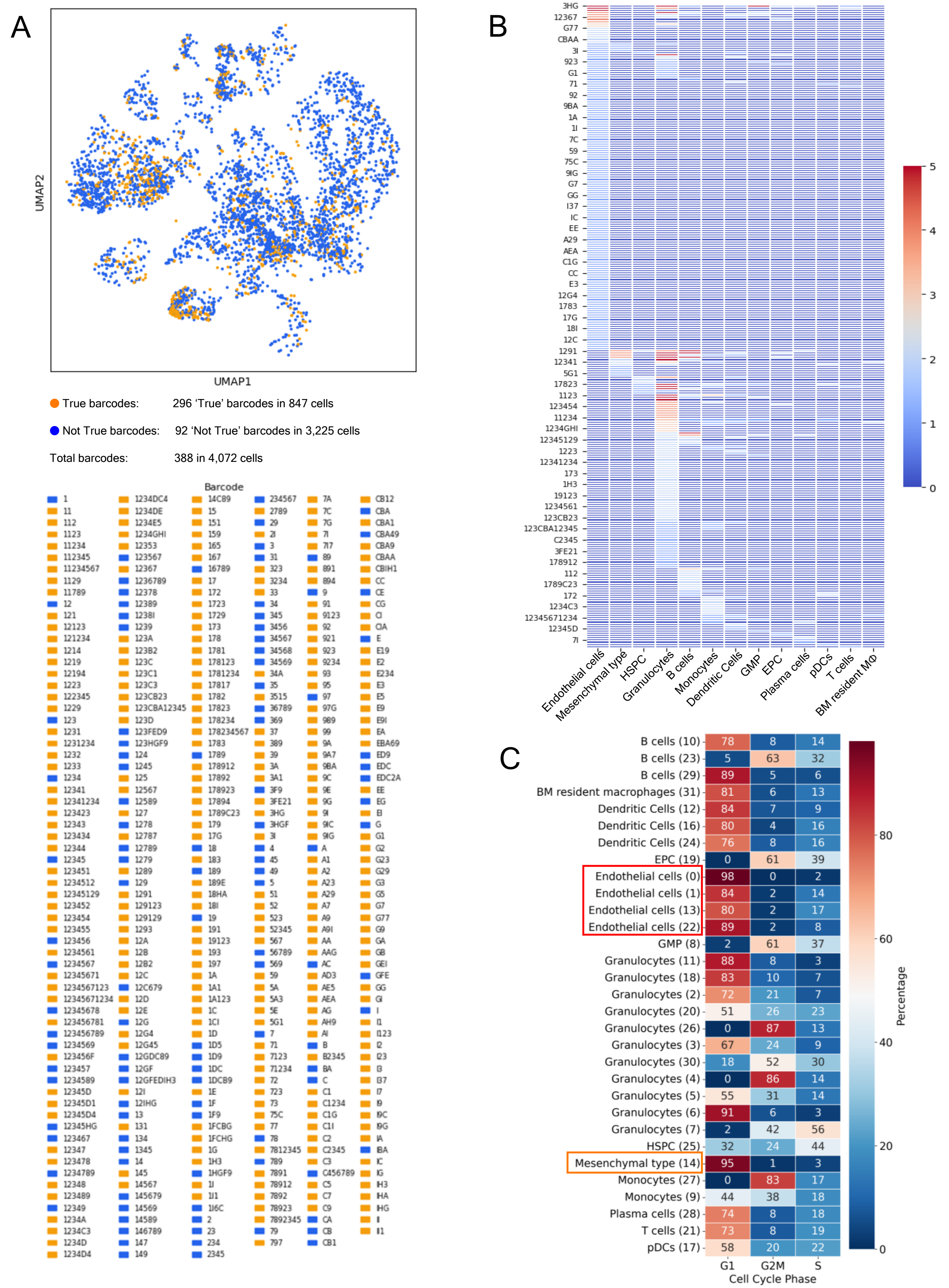

A
UMAP2
UMAP1
True barcodes: 296 ‘True’ barcodes in 847 cells
Not True barcodes: 92 ‘Not True’ barcodes in 3,225 cells
Total barcodes: 388 in 4,072 cells
Barcode
B
Endothelial cells
Mesenchymal type
HSPC
Granulocytes
B cells
Monocytes
Dendritic Cells
GMP
EPC
Plasma cells
pDCs
T cells
BM resident MΦ
C
B cells (10) 78 8 14
B cells (23) 5 63 32
B cells (29) 89 5 6
BM resident macrophages (31) 81 6 13
Dendritic Cells (12) 84 7 9
Dendritic Cells (16) 80 4 16
Dendritic Cells (24) 76 8 16
EPC (19) 0 61 39
Endothelial cells (0) 98 0 2
Endothelial cells (1) 84 2 14
Endothelial cells (13) 80 2 17
Endothelial cells (22) 89 2 8
GMP (8) 2 61 37
Granulocytes (11) 88 8 3
Granulocytes (18) 83 10 7
Granulocytes (2) 72 21 7
Granulocytes (20) 51 26 23
Granulocytes (26) 0 87 13
Granulocytes (3) 67 24 9
Granulocytes (30) 18 52 30
Granulocytes (4) 0 86 14
Granulocytes (5) 55 31 14
Granulocytes (6) 91 6 3
Granulocytes (7) 2 42 56
HSPC (25) 32 24 44
Mesenchymal type (14) 95 1 3
Monocytes (27) 0 83 17
Monocytes (9) 44 38 18
Plasma cells (28) 74 8 18
T cells (21) 73 8 19
pDCs (17) 58 20 22
G1 G2M S
Cell Cycle Phase
Percentage

Figure S5. Identification and distribution of 'True' Polylox barcodes across cell types. Related to Figure 5.
(A) UMAP plot showing the distribution of 'True' Polylox barcodes across the 34 Leiden-defined clusters identified in figure S4A. Cells containing 'True' barcodes (n = 847) are shown in orange; cells with 'Not True' barcodes (n = 3,225) are shown in blue. A total of 388 barcodes were detected, including 296 'True' (orange) and 92 'Not True' (blue), as listed below the UMAP plot.
(B) Heatmap displaying the distribution and abundance of 'True' Polylox barcodes across annotated cell types. Each row corresponds to a unique barcode (1 out of every 5 barcodes shown); color intensity represents the number of cells carrying that barcode within each listed cell type.
(C) Heatmap showing cell cycle phase distribution (G1, G2/M, S) across Leiden clusters identified in figure S4A. Color intensity and numerical values represent the percentage of cells in each phase within the indicated cell type.

Figure S6

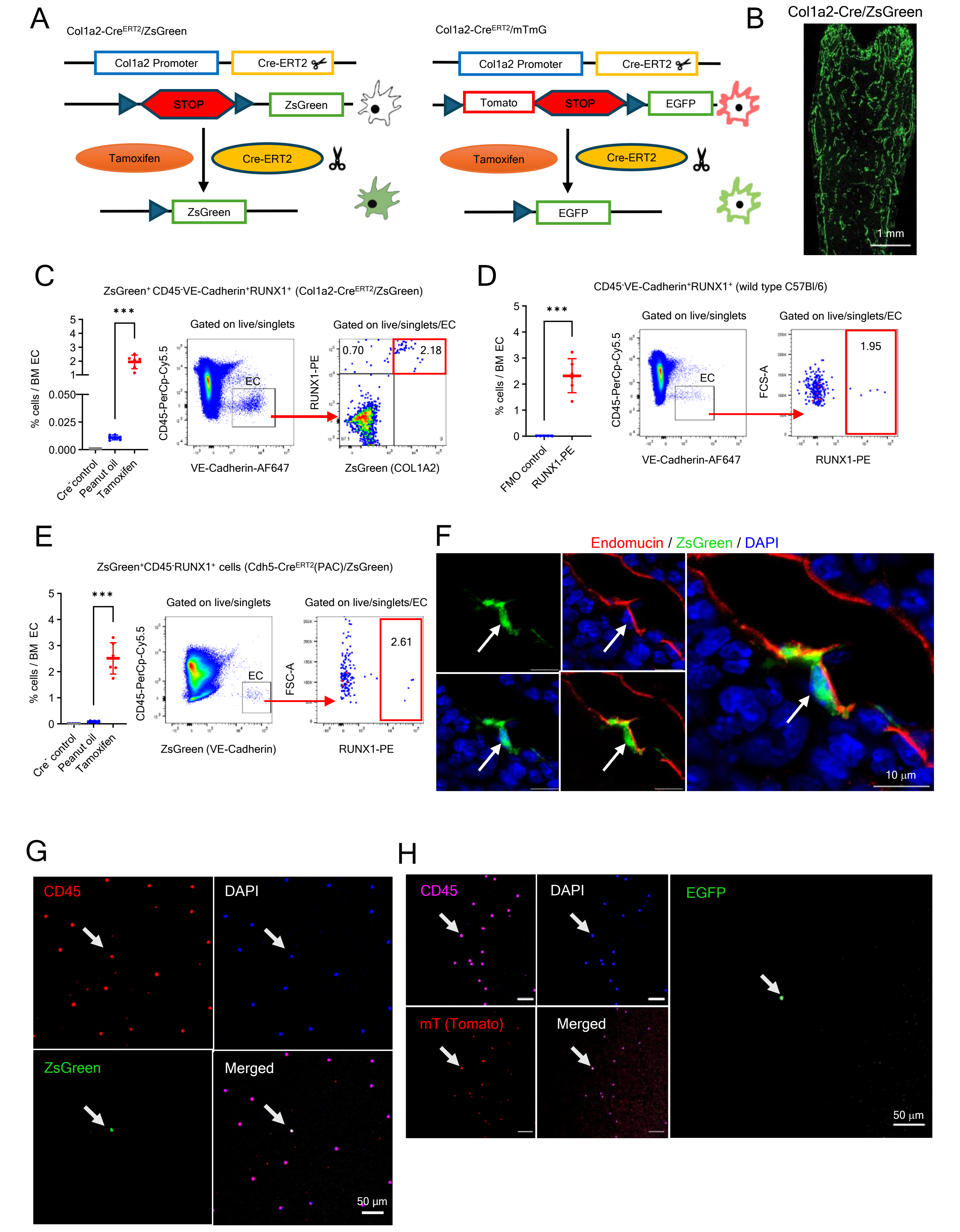

A
Col1a2-CreERT2/ZsGreen
Col1a2 Promoter
Cre-ERT2
STOP
ZsGreen
Tamoxifen
Cre-ERT2
ZsGreen
Col1a2-CreERT2/mTmG
Col1a2 Promoter
Cre-ERT2
Tomato
STOP
EGFP
Tamoxifen
Cre-ERT2
EGFP
B
Col1a2-Cre/ZsGreen
1 mm
C
ZsGreen+ CD45-VE-Cadherin+RUNX1+ (Col1a2-CreERT2/ZsGreen)
% cells / BM EC
Cre- control
Peanut oil
Tamoxifen
Gated on live/singlets
CD45-PerCp-Cy5.5
EC
VE-Cadherin-AF647
Gated on live/singlets/EC
RUNX1-PE
0.70
2.18
ZsGreen (COL1A2)
D
CD45-VE-Cadherin+RUNX1+ (wild type C57Bl/6)
% cells / BM EC
FMO control
RUNX1-PE
Gated on live/singlets
CD45-PerCp-Cy5.5
EC
VE-Cadherin-AF647
Gated on live/singlets/EC
FCS-A
1.95
RUNX1-PE
E
ZsGreen+CD45-RUNX1+ cells (Cdh5-CreERT2(PAC)/ZsGreen)
% cells / BM EC
Cre- control
Peanut oil
Tamoxifen
Gated on live/singlets
CD45-PerCp-Cy5.5
EC
ZsGreen (VE-Cadherin)
Gated on live/singlets/EC
FSC-A
2.61
RUNX1-PE
F
Endomucin / ZsGreen / DAPI
10 µm
G
CD45
DAPI
ZsGreen
Merged
50 µm
H
CD45
DAPI
EGFP
mT (Tomato)
Merged
50 µm

Figure S6. Characterization of Col1a2-tracked cell populations in BM and blood. Related to Figure 7.
(A) Schematic representation of the Col1a2 tracking lines.
(B) Representative confocal image of a BM section from a tamoxifen-treated Col1a2-Cre^{ERT2}/ZsGreen mouse, showing widespread distribution of $ZsGreen^{+}$ cells.
(C to E**)** Flow cytometric identification of $RUNX1^{+}$VE-$Cadherin^{+}CD45^{-}$ ECs in the BM of peanut oil-treated (n=6) and tamoxifen-treated (n=6) Col1a2-Cre^{ERT2}/ZsGreen adult mice; Cre^{-} mice (n=5) (C); WT C57Bl/6 mice (n= 6) and Fluorescence Minus One (FMO) control (n=5) (D); and Cdh5-Cre^{ERT2}(PAC)/ZsGreen mice treated with peanut oil (n=6) or tamoxifen (n=6); Cre^{-} mice (n=5)(E). Left panels: quantification of cells identified by the indicated gates as a percentage of total BM ECs; each dot represents one mouse (1 femur + 1 tibia). Middle and right panels: representative gating strategies.
(F) Representative confocal microscopy image of a BM section from a tamoxifen-treated Col1a2-Cre^{ERT2}/ZsGreen adult mouse showing a $ZsGreen^{+}$ $Endomucin^{+}$ cell lining a vascular structure (white arrows).
(G) Representative confocal image of a blood smear from a Col1a2-Cre^{ERT2}/ZsGreen mouse treated with tamoxifen showing the presence of a nucleate $CD45^{+}$ cell tracked by ZsGreen/Col1a2 fluorescence (pointed by the arrow).
(H) Representative confocal image of a blood smear from a Col1a2-Cre^{ERT2}/mTmG mouse treated with tamoxifen showing the presence of a nucleated $CD45^{+}$ cell tracked by EGFP/Col1a2 fluorescence (pointed by the arrow).
Dots represent individual mice. Data are shown as mean ± SD. ***$p < 0.001$ by Student's t test.

Figure S7

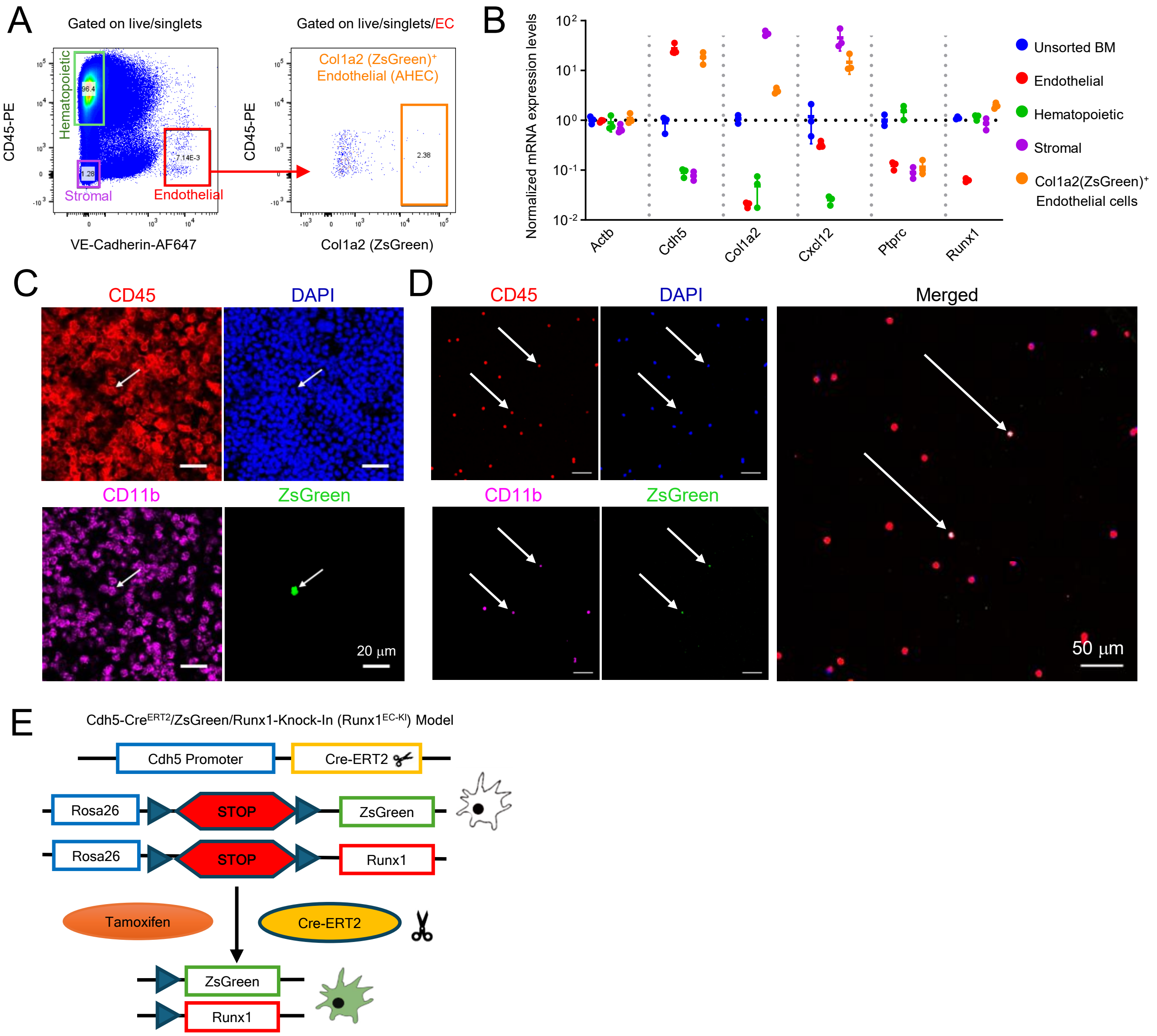

A
Gated on live/singlets
Gated on live/singlets/EC
CD45-PE
Hematopoietic
96.4
1.28
7.14E-3
Stromal
Endothelial
VE-Cadherin-AF647
Col1a2 (ZsGreen)+ Endothelial (AHEC)
2.38
Col1a2 (ZsGreen)
B
Normalized mRNA expression levels
Actb
Cdh5
Col1a2
Cxcl12
Ptprc
Runx1
Unsorted BM
Endothelial
Hematopoietic
Stromal
Col1a2(ZsGreen)+ Endothelial cells
C
CD45
DAPI
CD11b
ZsGreen
20 µm
D
CD45
DAPI
Merged
CD11b
ZsGreen
50 µm
E
Cdh5-CreERT2/ZsGreen/Runx1-Knock-In (Runx1EC-KI) Model
Cdh5 Promoter
Cre-ERT2
Rosa26
STOP
ZsGreen
Rosa26
STOP
Runx1
Tamoxifen
Cre-ERT2
ZsGreen
Runx1

Figure S7. Analysis and hemogenic potential of Col1a2-tracked adult BM ECs. Related to Figure 7.
(A) Representative FACS gating strategy used to isolate BM hematopoietic cells, ECs, stromal cells, and Col1a2-tracked ECs from tamoxifen-treated Col1a2-Cre^{ERT2}/ZsGreen mice.
(B) Gene expression profiling of unsorted BM and sorted BM populations defined in (A). Results from qRT-PCR are normalized by *Gapdh* and unsorted BM. Dots reflect experimental triplicates.
(C) Representative confocal image of a BM section from a transplant recipient showing a CD45^{+}CD11b^{+}ZsGreen^{+} cell (arrow), indicating hematopoietic derivation from transplanted ZsGreen^{+}VE-Cadherin^{+}CD45^{-} (Col1a2^{+}) cells.
(D) Representative image of a blood smear from a WT recipient mouse transplanted with ZsGreen (Col1a2)$^{+}$ BM ECs from Col1a2-Cre^{ERT2}/ZsGreen mice. ZsGreen tracked cells are pointed by the arrows.
(E) Schematic diagram of the Cdh5-Cre^{ERT2}/ZsGreen/Runx1-Knock-in (Runx1$^{EC\text{-}KI}$) mouse line used to trace endothelial cells with Runx1 expression induced upon tamoxifen treatment.
